# Antimicrobial Resistance in Nosocomial Isolates of Gram-Negative Bacteria: Public Health Implications in the Latvian Context

**DOI:** 10.3390/antibiotics10070791

**Published:** 2021-06-29

**Authors:** Nityanand Jain, Inese Jansone, Tatjana Obidenova, Raimonds Simanis, Jānis Meisters, Dagnija Straupmane, Aigars Reinis

**Affiliations:** 1 Department of Biology and Microbiology, Faculty of Medicine, Riga Stradiņš University, LV-1007 Riga, Latvia; aigars.reinis@rsu.lv; 2 Joint Laboratory, Pauls Stradiņš Clinical University Hospital, LV-1002 Riga, Latvia; inese.jansone@stradini.lv (I.J.); tatjana.obidenova@stradini.lv (T.O.); janis.meisters@stradini.lv (J.M.); dagnija.straupmane@stradini.lv (D.S.); 3 Department of Infectology, Faculty of Medicine, Riga Stradiņš University, LV-1007 Riga, Latvia; raimonds.simanis@stradini.lv

**Keywords:** AMR, antimicrobial resistance, antibiotics, nosocomial infection, gram-negative bacteria, ESBL

## Abstract

Antimicrobial resistance (AMR) is one of the most serious threats in modern medicine which requires the constant monitoring of emerging trends amongst clinical isolates. However, very limited surveillance data is available in the Latvian context. In the present study, we conducted a retrospective analysis of microbiological data from one of the largest public multispecialty hospitals in Latvia from 2017 to 2020. AMR trends for 19 gram-negative bacterial (GNB) genera were investigated. During the study period, 11,437 isolates were analyzed with *Escherichia* spp. (34.71%), *Klebsiella* spp. (19.22%) and *Acinetobacter* spp. (10.05%) being the most isolated. Carbapenems like Meropenem and Ertapenem were the most effective against GNBs (3% and 5.4% resistance rates, respectively) while high resistance rates (>50%) were noted against both Ampicillin and Amoxicillin/Clavulanic acid. *Enterobacter* spp. and *Klebsiella* spp. showed a significant increase in resistance rate against Ertapenem (*p* = 0.000) and Trimethoprim-Sulfamethoxazole (*p* = 0.000), respectively. A decrease in the prevalence of Extended-Spectrum Beta-Lactamase positive (ESBL+) Enterobacterales was noted. Despite the lower prescription levels of the penicillin group antimicrobials than the European average (as reported in ESAC-Net Surveillance reports), GNBs showed high average resistant rates, indicating the role of ESBL+ isolates in driving the resistance rates. Constant and careful vigilance along with proper infection control measures are required to track the emerging trends in AMR in GNBs.

## 1. Introduction

Antimicrobial resistance (AMR) represents a global crisis that endangers the efficacy of the antibiotics, and consequently, also the efficacy of the treatment provided. According to WHO, AMR is defined as the resistance in different types of microorganisms against various agents like antibacterial, antiviral, antiparasitic and antifungal drugs [1]. Under normal conditions, AMR is acquired naturally, however, acquisition rates can be accelerated by antibiotic overuse, inappropriate prescription and polypharmacy as well as a lack of new drug development by the pharmaceutical industry, which is precipitated with strict regulations [2]. The problem gets amplified further when one considers the aging demographics in most of the developed countries and the increase in immunocompromised patients [3]. Another challenge associated with AMR is the increasing incidence of HAIs (hospital-acquired infections) or nosocomial infections, which are defined as infections that occur more than 48 h post-admission in a hospital or medical care center and are caused by a myriad of microbes ranging from bacteria to fungi to viruses [4]. Inside the hospital setting, however, intrinsic factors like clustering of highly vulnerable patients, extensive use of invasive procedures, and high rates of antibiotic use are the concerning factors contributing to the development of AMR in HAIs [5]. All these factors ultimately lead to a huge financial burden on both the patients and hospitals besides the prolongation of hospitalization time. 

Amongst the commonly isolated HAIs, gram-negative bacteria (GNBs) are of particular concern, especially given the significantly higher rate of associated complications [6]. Further, previous studies have indicated that GNBs are becoming resistant to most of the antibiotic drug options available, thereby creating situations that may mimic the pre-antibiotic era [2,5,7]. Hence, it becomes crucial to overcome and tackle the dual AMR-HAI crisis by understanding the patterns in AMR acquisition and spread to adjust our antimicrobial prescriptions accordingly. One of the tools available for such surveillances are Antibiograms, which show the development of antibiotic resistance and susceptibility over continuous time periods [8]. Studying such graphs helps us to compile and profile the resistance rates of specific bacteria to individual antimicrobial agents which are routinely tested and prescribed in clinics, thus empowering the clinicians with a method of more targeted and effective selection of empiric antibiotics [8]. Additionally, such studies can act as a cross-sectional audit of antimicrobial use in hospitals. 

Within the GNBs identified as HAIs, ESKAPE pathogens are the most worrisome and burdensome. They account for a majority of the reported nosocomial infections and can escape the bactericidal effects of the commonly used antibiotics [9,10]. The term ESKAPE encompasses six pathogens–two gram-positive (*Enterococcus faecium* and *Staphylococcus aureus*) and four gram-negative (*Klebsiella pneumoniae, Acinetobacter baumannii, Pseudomonas aeruginosa*, and *Enterobacter* species). Other common and emerging species of concern include the ones belonging to the order Enterobacterales like *Citrobacter* spp., * Escherichia* spp., *Morganella* spp., *Proteus* spp. and *Serratia* spp. The members of this order can produce antibiotic inactivating enzymes (especially β-lactamases) that make them resistant to a variety of different classes of antibiotics. Outside of this order, well-known nosocomial agents are *Campylobacter* spp., *Neisseria* spp., *Moraxella* spp. and *Burkholderia* spp, while an increasing number of cases are being reported for the others like *Achromobacter* spp., *Aeromonas* spp., *Bacteroides* spp., *Chryseobacterium* spp., *Prevotella* spp. and *Stenotrophomonas* spp.

Amongst the commonly tested and prescribed antibiotics, penicillin group antibiotics are the first choice to treat gram-negative bacterial infections. Ampicillin (AMP) works by binding and inactivating receptors called membrane-bound penicillin-binding proteins (PBPs), which arrest cell growth and formation of the cell wall (via inhibition of peptidoglycan synthesis) [11]. Amoxicillin-clavulanic acid (AMC) is a combination of amoxicillin, a broad spectrum β-lactam penicillin derivative that works in a similar way to AMP, and clavulanic acid, a β-lactamase inhibitor (an enzyme secreted by bacteria as a defense against antibiotic), which protects amoxicillin from degradation [12,13]. Like AMC, piperacillin-tazobactam (TPZ) represents a β-lactam/β-lactamase inhibitor combination.

Aminoglycosides like amikacin (AMK) and gentamycin (GEN) are the next choice and are often used in combination with penicillin to treat severe nosocomial infections. They block protein synthesis by binding to 30S bacterial ribosomal subunits, which lead to their bactericidal effects [14,15]. Very similar to aminoglycosides, amphenicols like chloramphenicol (CHL) inhibit protein synthesis via binding to a 50S bacterial ribosomal subunit [16]. Quinolones like Ciprofloxacin (CIP) are used in patients predisposed to gram-negative bacteria (immunosuppression, prolonged hospitalization, co-morbidity etc.), which inhibits DNA replication by blocking the action of bacterial DNA topoisomerase and DNA-gyrase [17,18]. Trimethoprim-sulfamethoxazole (SXT), another commonly used antibiotic agent, works by blocking the bacterial biosynthesis of essential nucleic acids and proteins [19].

Finally, the excessive use of antibiotics belonging to the carbapenem and 3rd generation cephalosporin classes, increased in the last years the AMR trends which have been and still are a cause of concern worldwide. Carbapenems like Ertapenem (ETP), Imipenem (IPM) and Meropenem (MEM) belong to β-lactam class of antibiotics, which work by binding and inhibiting PBPs. However, unlike penicillin, carbapenems are relatively resistant to degradation by β-lactamases, which makes them extremely useful in the case of multi-drug resistance gram-negative bacteria [20]. Third generation cephalosporins like ceftazidime (CAZ) and cefotaxime (CTX) also bind to PBPs and inhibit cell wall formation, which leads to cell death due to osmotic lysis, and, like carbapenems, they are relatively resistant to the action of β-lactamases [21].

Hence, in the present study, we aimed to collect and analyze AMR data along with reporting the frequency and source of HAIs caused by 19 different GNB genera, with the intention to improve the overall public health management of GNB-associated HAIs in Latvia and its implications on antimicrobial usage patterns across Latvian healthcare centers. The present study was conducted from 2017 to 2020 at the Joint Laboratory of Pauls Stradiņš Clinical University Hospital (PSCUH), Riga, Latvia, which is one of the largest multispecialty hospitals in Latvia. Furthermore, our laboratory since 2004, has been providing AMR data to EARS-Net (European Antimicrobial Resistance Surveillance Network), which monitors AMR data on seven bacterial pathogens commonly causing infections in humans [22] along with providing reference services for other regional microbiological laboratories.

## 2. Results

### 2.1. Annual Prevalence of Various Gram-Negative Bacteria

During the study period, a total of 11,437 (2705 in 2017; 2812 in 2018; 2993 in 2019 and 2927 in 2020) GNB samples were isolated, spread over 19 different GNB genus (Table 1). The most isolated species were *Escherichia* spp. (34.71%) followed by *Klebsiella* spp. (19.22%) and *Acinetobacter* spp. (10.05%). Together, these three genera accounted for more than 60% of the total isolates. The distribution of the isolates based on genus, species, department, and sample type is shown in Supplementary Material File S1.

### 2.2. Weighted Cumulative Antibiotic Resistance Rates and Trends over the Study Period

**Figure 1:**
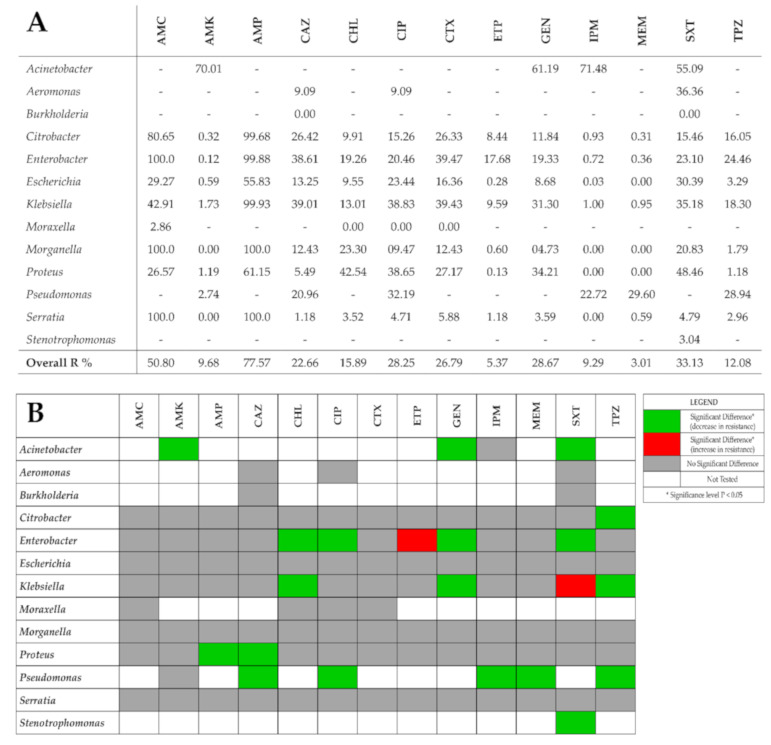
(**A**) Average resistance rates against tested antimicrobial agents and GNBs studied. Overall R% indicates the weighted average of resistance rates taking into count the number of isolates and the relevant resistance rates. (**B**) Trends in bacterial resistance to tested antibiotics across the study period (2017 vs 2020) using χ^2^ test. AMC—amoxicillin-clavulanic acid; AMK—amikacin; AMP—ampicillin; CAZ—ceftazidime; CHL—chloramphenicol; CIP—ciprofloxacin; CTX—cefotaxime; ETP—ertapenem; GEN—gentamicin; IPM—Imipenem; MEM—meropenem; SXT—trimethoprim-sulfamethoxazole; TPZ—piperacillin-tazobactam.

Over the study period, the GNBs showed high resistance against penicillin group (amoxicillin/clavulanic acid–50.80%; ampicillin–77.57%) and were found to be susceptible to carbapenems like imipenem, meropenem and ertapenem (weighted overall resistance < 10%). However, the GNBs were found to be less resistant to piperacillin/tazobactam (12.08%). Weighted resistance to aminoglycosides varied from 10% against amikacin to almost 30% against gentamicin. GNBs showed about 25% resistance rate against cephalosporins like cefotaxime and ceftazidime (Figure 1A). Similar resistance rates were observed against ciprofloxacin (fluoroquinolone). Resistance to chloramphenicol was about 16%, while resistance was more than double against trimethoprim/sulfamethoxazole (33.13%). The yearly resistance rates are summarized in Supplementary File S2.

The χ^2^ test was used to analyze the antimicrobial resistance rates for each bacterial genus in the first (2017) and last (2020) study year as shown in Figure 1B. In 90 out of 111 (81.1%) bacteria–antimicrobial agent combinations, no significant change in resistance rates were observed. Nineteen (17.1%) bacteria–antimicrobial agent combinations showed a significant decrease in resistance rates. Only two combinations showed a significant increase in resistance rates—*Enterobacter* spp. showed a significant increase of 18.4% in resistance rates against Ertapenem (ETP) (*p* = 0.000; Φ = 0.241) while *Klebsiella* spp. showed a significant increase of 12.4% in resistance rates against Trimethoprim-Sulfamethoxazole (SXT) (*p* = 0.000; Φ = 0.124).

### 2.3. Overall Weighted Multiple Antibiotic Resistance (MAR) Index

**Figure 2:**
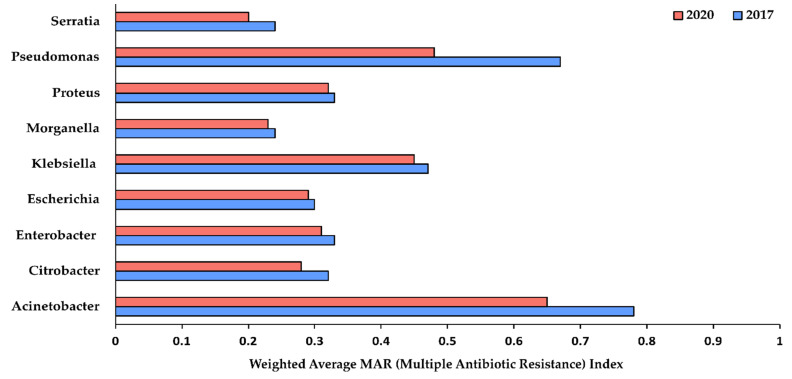
Weighted average of MAR (multiple antibiotic resistance) index for different gram-negative nosocomial bacterial genera showing resistance to various tested antibiotics. MAR ≥ 0.2 indicates isolates originated from the source having a high-risk of antimicrobial contamination.

We found that isolates representing nine genera of the GNBs showed resistance against at least two or more of the tested antimicrobial agents. Isolates from *Enterobacter* spp., *Serratia* spp. and *Acinetobacter* spp., showed the highest number of isolates showing resistance against at least two of the tested antimicrobial agents (91.81%, 87.10% and 82.33%, respectively in 2017 and 89.18%, 85.71% and 69.08%, respectively in 2020) (Supplementary File S3). Since MDR testing was not performed, we analyzed carbapenem-resistance (CR) as a relative measure of MDR (multi-drug resistance) estimation. We found that less than 10% of the all isolates (Figure 1) showed carbapenem-resistance (CR), probably indicating the low prevalence of MDR isolates in our hospital. Furthermore, the weighted average MAR Index was calculated to understand the levels of risk of antimicrobial contamination. *Acinetobacter* spp. and *Pseudomonas* spp. isolates were found to be resistant to more than 70% of different antimicrobials tested in 2017, which dropped to 60% and 50% respectively (Figure 2).

### 2.4. Extended-Spectrum Beta-Lactamases (ESBL)-Producing Enterobacterales

**Figure 3:**
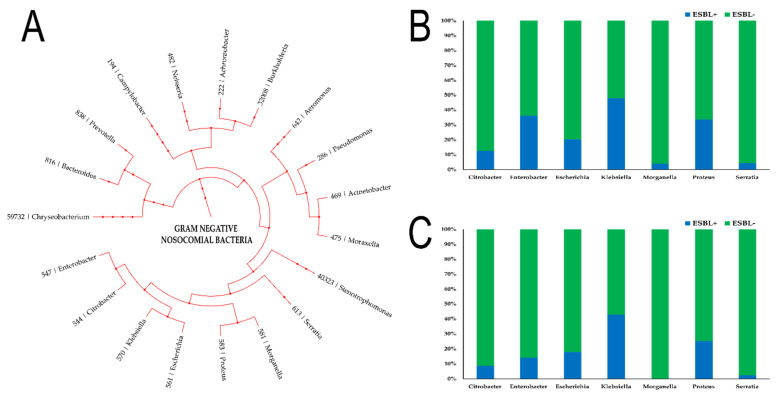
Percentage of isolates that were identified as Extended-Spectrum Beta-Lactamases positive (ESBL+). (**A**) Phylogenetic tree showing the phylogenic relationship of the different gram-negative bacteria studied in the present study along with their respective NCBI Taxon ID; (**B**) Percentage (%) of isolates that were ESBL+ among different genera of Enterobacterales in 2017; (**C**) Percentage (%) of isolates that were ESBL+ among different genera of Enterobacterales in 2020.

Overall, our results showed that there was a decrease in the number of ESBL+ Enterobacterales in 2020 when compared with the start of the study period in 2017. About 48% of all *Klebsiella* spp. isolates were found to be ESBL+ in 2017 while the number decreased to 43% in 2020 (Figure 3). Similarly, about 35% of *Proteus* spp. and *Enterobacter* spp. isolates were ESBL+ in 2017, which decreased to 25% and 14% respectively in 2020. For both *Morganella* spp. and *Serratia* spp. isolates, less than 5% isolates were found to be ESBL+ in 2017 and 2020.

### 2.5. Consumption Patterns of Antimicrobials in the Hospital Sector (2017–2019)

**Figure 4:**
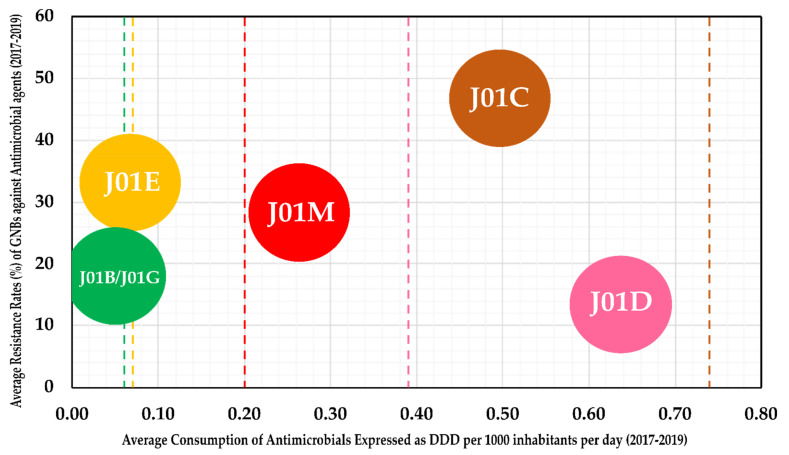
Average consumption rates of antimicrobials expressed as DDD (defined daily dose) per 1000 inhabitants per day (x-axis) against the average resistance rates of GNBs obtained in the present study (y-axis). Antimicrobials were classified based on WHO’s Anatomical Therapeutic Chemical Classification System—J01B includes CHL; J01G includes AMK and GEN; J01E includes SXT; J01M includes CIP; J01C includes AMC, AMP and TPZ; J01D includes CAZ, CTX, ETP, IPM and MEM. The colored lines indicate the respective group average for EEA/EU (European Economic Area/European Union).

For understanding the consumption patterns of antimicrobials in the hospital sector in Latvia, we analyzed the data from ESAC-Net (European Surveillance of Antimicrobial Consumption Network) Surveillance reports from 2017 to 2019 (the report for 2020 is yet to be published). Accordingly, J01M (Quinolones) and J01D (other β-lactams) were found to be prescribed at a higher rate in Latvia than the EEA/EU (European Economic Area/European Union) averages for these groups (Figure 4). However, the resistance of GNBs against both of these groups remained below 30%. Interestingly, although β-lactams and penicillin (J01C) group antimicrobials were prescribed at a lesser rate than the EEA/EU average, the average resistance rates remained at around 50%, indicating the role of ESBL+ isolates in driving high resistance rates. The average consumption rates of J01E (sulfonamides and trimethoprim) and J01B/J01G (Amphenicols and Aminoglycosides) antimicrobials in the hospital settings in Latvia remained close to European averages, with a low overall resistance against the two antibacterial classes (<35%).

## 3. Discussion

According to previously published estimates, close to 3.8 million patients contract nosocomial infections each year in the European Union (EU) [23] with close to 33,000 yearly reported deaths that can be attributed to infections that were caused by bacteria that were resistant to antimicrobials [22]. This dual HAI-AMR threat adds a substantial financial burden onto the already overstretched health care resources. To treat such infections, when the first- or second-line antimicrobials fail, healthcare professionals usually resort to a use of the last-line antimicrobials, which are generally expensive and are associated with greater side effects [24]. However, despite the availability of such last resort antimicrobials, in most cases the patients require significantly longer hospital stays, more doctor’s visits, and suffer from associated psychological and health associated recuperations whilst experiencing a higher incidence of long-term disability [2,25]. Hence, proper management and surveillance become essential to tackle the problem of HAI-AMR.

### 3.1. Significant Findings of the Present Study (Summarized Report)

Our study reports for the first time in our knowledge, a detailed surveillance report for 19 different GNBs, which are usually identified as a cause of nosocomial infections in the Latvian context. Several new findings were revealed by the present study, which is particularly worrisome. Firstly, on average, 1 in every 17 (5.8%) patients who were admitted in the inpatient departments suffered from a GNB-associated nosocomial infection over the years. Secondly, the GNBs showed high resistance to commonly used oral antibiotics including ampicillin (AMP) and amoxicillin/clavulanic acid (AMC). Thirdly, although statistically insignificant, there is an increasing trend of AMR towards the broad-spectrum antibiotics including Imipenem and other β-lactams. Fourthly, there has been increasing resistance in *Klebsiella* spp. and *Enterobacter* spp. isolates against second-line or last resort antimicrobials. Finally, there were higher national rates of consumption of Quinolones (J01M) and other β lactams (J01D) in the hospital settings when compared with the EEA/EU average.

However, our findings also indicate some encouraging trends in HAI-AMR that need to be pursued further to tackle the problem of AMR. Firstly, there has been a decrease in the number of ESBL+ isolates over the years. This decrease is crucial, since these GNBs produce enzymes called beta-lactamases, which breakdown the beta-lactam rings in antibiotics like penicillin and amoxicillin, rendering the antibiotics useless against infections [26]. This is evident in the case of *Proteus* spp. Isolates, as the number of ESBL+ isolates decreased from 2017 to 2020, the AMR against ampicillin (AMP) also decreased significantly (*p* = 0.039; Φ = 0.113). Secondly, apart from *Klebsiella* spp. and *Enterobacter* spp. isolates, other GNBs showed either no significant trend changes or rather showed a significant decrease in AMR trends. This is very crucial, especially in the context of members of genera *Acinetobacter* spp. and *Pseudomonas* spp. since both are frequently known to cause life-threatening conditions that are difficult to treat due to their intrinsic resistance to many antimicrobial agents.

Nonetheless, in comparison with the EU/EEA average, the AMR rates remained high in Latvia. For example, the EU AMR average against Piperacillin-Tazobactam for *Pseudomonas* spp. isolates were around 16.7% from 2017 to 2019, whilst our isolates showed almost double AMR rates averaging around 31% (Supplementary File S2) [22]. A similar situation was noted for carbapenems with resistance in our isolates around 28% against the European average of 17%. For *Acinetobacter* spp. isolates, the situation is even worse. Whilst the EU average resistance rates were about 32.5% (2017–2019) against carbapenems, our samples showed an average resistance of 72%. We found that the resistance rates are 2- to 3-fold higher in our isolates than the EU average for *Acinetobacter* spp. against aminoglycosides like gentamycin and amikacin.

Apart from the traditional GNBs, species like *Escherichia* spp., *Klebsiella* spp., *Pseudomonas* spp., *Acinetobacter* spp., *Enterobacter* spp. and many other GNBs, genera are becoming an increasing concern in the hospital setting. GNBs like *Citrobacter* spp., *Morganella* spp., *Serratia* spp. and *Stenotrophomonas* spp. accounted for about 10% of the total GNB isolates in our study, indicating the need for increased reporting and surveillance of these genera as well. Furthermore, despite the low prevalence rates of ESBL+ isolates of *Citrobacter* spp., *Morganella* spp. and *Serratia* spp., the AMR against ampicillin (AMP) and amoxicillin/clavulanic acid (AMC) remained very high (above 80%). However, they showed minimal resistance to aminoglycosides, which can serve as a potential treatment avenue.

### 3.2. ESKAPE Pathogens–AMR in Klebsiella spp.

Members of this genus are well known to clinicians, both in the outpatient and inpatient setting. They are the causative agents behind community-acquired pneumonia, especially in chronic alcoholics [27]. However, the majority of the infection cases are rather associated with hospitalization. As an opportunistic pathogen, the two most important members of the genus—*K. pneumoniae* and *K. oxytoca*—are commonly found to target patients with underlying conditions like diabetes mellitus (DM) or chronic obstructive pulmonary disease (COPD) [28]. Blood products, contaminated medical equipment, the gastrointestinal tract of patients and the hands of hospital personnel represent the primary reservoirs and transmission source for *Klebsiella* species [29,30]. Certain medical procedures like an endoscopy can also lead to the transmission of the bacteria [31]. While *K. pneumoniae* is commonly associated with urinary tract infections (UTI), bronchopneumonia, soft tissue infections, and bacteremia, *K. oxytoca* is additionally associated with colitis and infective endocarditis [32]. The situation is extremely worrisome, given the frequent isolation of the *Klebsiella* species from the neonatal departments and wards.

In our study, roughly 15% of the *Klebsiella* spp. isolates were identified as *K. oxytoca*, while the vast majority (approximately 80%), were identified as *K. pneumoniae*. About a third of the samples were collected from urine specimens, followed by bronchoalveolar lavage (20%) and blood specimens (7%). Based on departments, intensive care, lung diseases and thoracic surgery, and nephrology accounted for close to 45% of the isolates. On the other hand, the neonatal department reported a rather lower caseload (close to 1.5%) in the study period (Supplementary File S1). Two major mechanisms have been reported to be responsible for inferring antimicrobial resistance to *K. pneumoniae*. The first being production of ESBL and the second being point mutations in genes encoding penicillin-binding proteins (PBPs) [33,34]. Furthermore, it has been shown that indiscriminate use of antibiotics upregulates the expression of ESBL [35]. We found that >40% of the isolates were ESBL+ with isolates showing complete resistance against ampicillin (average 99.93%).

Another resistance mechanism described is against quinolones (J01M; ciprofloxacin). Point mutations in DNA gyrase genes gyrA and gyrB along with topoisomerase IV genes parC and parE are thought to be the main mechanisms against quinolone resistance [36]. Although no significant change in resistance against CIP was noted, a downward trend was still noted with an average resistance rate of around 38%, indicating a relatively moderate prevalence of such mutations in the isolates. Additionally, a significant reduction in AMR rates was noted against GEN, TPZ and CHL. The % of carbapenem resistant isolates also remained low (<10% for ETP; <1% for IPM and MEM). However, more than a third of the isolates were found to be resistant against third generation cephalosporins like CTX and CAZ. Furthermore, the weighted MAR index remained close to 0.5, indicating that the isolates were collected from departments with a high antimicrobial use and around 53% of the isolates showed resistance against at least two of the tested antimicrobial agents (Supplementary File S3).

### 3.3. ESKAPE Pathogens—AMR in Acinetobacter spp.

This genus represents a heterogeneous group of ubiquitous free-living saprophytes that are part of the normal flora of the skin, mucous membranes, pharynx, and human respiratory secretions [37,38,39]. However, members of the genus have also been isolated from the forehead, conjunctiva, hand, vagina, perineum, axillae, groin, and toe webs in healthy individuals [40]. Due to their ability to survive dry conditions over long periods, they are frequently isolated from reusable medical equipment like ventilator tubing, arterial pressure monitoring devices, humidifiers, washbasins, plastic urinals, respirometers, the skin of healthcare personnel and bed linens and mattress [41,42]. We found that most of the specimens were isolated from bronchoalveolar lavage, tracheal aspirate, wound site and urine samples (Supplementary File S1). *A. baumannii* infections usually involve organ systems that contain high levels of fluids like the urinary and respiratory tract and peritoneal cavity [39]. It is frequently associated with hospital-acquired pneumonia with an isolate frequency of around 3–5% in ICUs (intensive care units) [43]. This is interesting, since we recorded more than a 10× isolation rate of around 35% in ICUs. *A. baumannii* has also been associated with bacteremia, UTI, trauma and wound infections, meningitis, and endocarditis [39]. About 10% of our isolates were collected from the neurology department, along with 15% from the cardiology department, which makes it essential to control the spread of *Acinetobacter* species.

Species in this genus have been labeled as “naturally transformable” due to their remarkable capacity for the acquisition of foreign genetic material including AMR genes [44]. Several mechanisms of gene transfer have been described, including transformation [45], conjugation [46], transduction [47] and mobile genetic elements like plasmids, transposons and integrons [48]. Resistance to AMK and GEN (aminoglycosides) is usually due in the presence of gene coding for aminoglycoside-modifying enzymes (AME) in integrons [38], while resistance to CIP is due to mutations in the gyrA and parC genes. While resistance against CIP was not tested, resistance against AMK and GEN remained very high, with AMR rates of about 70% and 60%, respectively. Carbapenem-resistant *A. baumannii* is a major concern, with the WHO (World Health Organization) putting it in the priority category against which research and development of new antibiotics is critical [49]. Production of carbapenemase, altered function of membrane-associated proteins and activation of drug efflux pumps have been described as the resistance mechanism against carbapenems [50]. Resistance rates against IPM remained very high (>70%) amongst our isolates. Finally, the weighted MAR index remained one of the highest in *Acinetobacter* spp., indicating that the isolates were resistant to most of the tested antimicrobial agents.

### 3.4. ESKAPE Pathogens–AMR in Pseudomonas spp.

Another frequent and difficult-to-treat causative agent, *Pseudomonas* spp., are known to colonize moist dark places like medical ventilators, oxygen respirators, humidifiers, sinks, taps, toilets, and dialysis machines [51,52]. In the hospital environment, *Pseudomonas* spp. are commonly identified as causative agents of bacteremia, pneumonia, urosepsis, and wound infection including the secondary infection of burns [53]. Multiple risk factors, including chemotherapy-induced neutropenia, surgical incisions, insertion of urinary and vascular catheters, endotracheal tubes, multiple organ injury and failure along with underlying comorbidities (like DM, COPD, cystic fibrosis etc.), have been reported [52,53]. Our analysis showed that about a third of the specimens were isolated from urine samples followed by wound site, ulcers and bronchoalveolar lavage. Based on departments, the highest caseload was in the Nephrology department, followed by lung disease and thoracic surgery and ICU.

Generally, the antibiotic resistance mechanisms in this genus are classified as intrinsic (low outer membrane permeability, efflux pumps and production of antibiotic-inactivating enzymes), acquired (mutational changes and horizontal gene transfer) and adaptive (formation of biofilms) [54,55,56,57]. This makes the genus resistant to a wide variety of antibiotic classes, including aminoglycosides, quinolones, and β-lactams [54] and earns it (carbapenem resistant *P. aeruginosa*) a place right with *A. baumannii* in the WHO class of priority category against which new antibiotics are critically needed [49]. Our isolates showed about 20–30% resistance rates against 3rd generation cephalosporins, quinolones and carbapenems with a significant decrease in resistance rates in 2020 compared to 2017. Resistance against aminoglycosides (AMK) remained very low (around 2%), which could be used for the treatment of pseudomonal infections. Furthermore, amongst ESKAPE pathogens, *Pseudomonas* spp. and *Enterobacter* spp. isolates remained relatively less commonly isolated. Nonetheless, the weighted MAR index remained close to 0.5 in 2020, indicating resistance against half of the tested antimicrobial agents.

### 3.5. ESKAPE Pathogens–AMR in Enterobacter spp.

Finally, in terms of the last of the Gram-negative ESKAPE pathogens, members of this genus are responsible for causing nosocomial UTI, lower respiratory infections, soft tissue infections, osteomyelitis, surgical site infections, bacteremia and endocarditis, among many others [58]. Some members of the genus are known to be part of the normal microflora of gastrointestinal and respiratory tracts [59,60]. Transmission in hospital departments is mainly due to contaminations caused by intravenous injection fluids, blood products, stethoscopes, cotton swabs, and colonized hands of healthcare professionals [61]. Amongst the known 22 species, *E. cloacae* remains the most isolated and studied in the context of nosocomial infections. Indeed, *E. cloacae* remained the most isolated species (>85%) in our samples followed by *E. aerogenes* and *E. asburiae* (Supplementary File S1). Urine, bronchoalveolar lavage and wound site remained the most common samples from which *Enterobacter* species were isolated (>50%). Nephrology, lung diseases and thoracic surgery along with ICU remained the departments with the highest caseload.

*Enterobacter* spp. showcase intrinsic resistance to penicillin, amoxicillin-clavulanate, first-generation cephalosporins, and cefoxitin due to their ability to produce AmpC β-lactamase [62]. It was evident in our isolates as well, achieving close to 100% resistance rates against AMC and AMP. Although the prevalence of ESBL+ *Enterobacter* spp. decreased from close to 35% in 2017 to about 15% in 2020, the resistance against beta-lactams did not change, indicating the presence of certain acquired resistance mechanisms in the isolates. Resistance to carbapenems is conferred due to its carbapenemase production capability. Although resistance against ETP remained close to 19% (a significant increase from 2017 was noted), resistance against MEM and IPM remained below 1%, indicating low prevalence rates of carbapenem-resistant *Enterobacter* spp. and over-dependence on ETP for treatment of enterobacterial infections. Weighted MAR index also remained relatively low at 0.3, indicating that the isolates showed resistance against 30% of the tested antimicrobial agents. Resistance against 3rd generation cephalosporins showed no significant trend change, with average resistance rates at around 40%.

### 3.6. AMR Rates in Other Enterobacterales Order Members (CESP and beyond)

Enterobacterales include a large order of ESBL-producing gram-negative bacteria which have been identified as causative nosocomial agents. CESP represents a subgroup within this order which are known to produce AmpC-encoded β-lactamases. The subgroup includes *Citrobacter* spp., *Enterobacter* spp., *Serratia* spp. and *Providencia* spp. (data not shown). Outside this subgroup, *Morganella* spp. and *Pseudomonas* spp. are also known to produce AmpC-encoded β-lactamases [63]. *Escherichia* spp. and *Proteus* spp. were the remaining order members that were included in the present study.

The genus Citrobacter is represented by more than ten species that are known to reside inside the intestines of animals and humans. Of these species, *C. freundii* is associated with gastroenteritis, UTI, neonatal meningitis, and septicemia, while *C. koseri* causes neonatal meningitis and brain abscesses [64,65]. *Citrobacter* spp. are often considered as emerging nosocomial agents, especially in developing countries. Invasive procedures like catheterization or genitourinary instrumentation, along with immune suppression and prolonged hospitalization durations, put the patients at risk of infection [66]. In our hospital, the *Citrobacter* spp. caseload was around 3% of all the GNB genera studied. Although *C. freundii* and *C. koseri* were the most isolated species, there was a more than 10× increase in isolates of *C. braakii.* Most of the specimens were isolated from urine, sputum, wound site, bronchoalveolar lavage and stool samples. Nephrology, neurology, and general surgery, along with thoracic surgery and cardio surgery departments, showed the highest incidence of the isolates. Like other genera in Enterobacterales, resistance against AMP and AMC remained high (>80%). No significant change in AMR rates was observed, except a significant decrease against TPZ during the study period. Resistance against carbapenems remained below 1% for IPM and MEM and around 8% against ETP, while resistance against third generation cephalosporins remained at around 26%.

Infections caused by *Serratia* spp. are rarely community acquired; however, they are increasingly being recognized as a major cause of nosocomial infections. Of the 14 known species, *S. marcescens, S. liquefaciens* and *S. odorifera* are the most known and clinically implicated [67,68]. As an opportunistic bacterium, it is generally known to cause bacteremia, UTI and respiratory tract infections, with patients undergoing invasive procedures like indwelling and intravenous catheterization and respiratory intubation [69]. Our results indicate that there was a decrease in the % of *S. marcescens*, while an increase in *S.liquefaciens* was noted. Bronchoalveolar lavage, tracheal aspirate, urine, and sputum samples were the most common isolation samples. Thoracic surgery, ICU, cardiology, and vascular surgery departments remained the most burdened with the caseload. Resistance to penicillin remained at 100%, while resistance against carbapenems and cephalosporins remained below 5%. Resistance against other classes as well remained below or around 5%. ESBL+ samples were rarely isolated (5% in 2017 which decreased to 3% in 2020).

*Morganella morganii* is an unusual opportunistic bacterium that has been known to rarely cause nosocomial infection [70]. It is known to cause UTI and postoperative wound infections along with sepsis, abscess, purple urine bag syndrome, chorioamnionitis, and cellulitis [71]. It is often considered a bacterium with increased levels of resistance and virulence. Old age, concomitant bacteremia, prolonged hospitalization, recent surgery, and concurrent antibiotic use have been established as risk factors for *Morganella* infection [72]. Furthermore, transmission from animals (like dogs, snakes) via bites or scratches to an immunocompromised host is also seen as a risk factor [71,73]. Administration of such patients should not be overlooked and could be potentially dangerous, given the associated high mortality rates [71]. We isolated most specimens from urine and trophic ulcer samples (approx. 40%) with nephrology and general surgery departments showcasing the highest caseload. The bacterium has an intrinsic resistance to penicillin, while it remains generally susceptible to aminoglycosides, quinolones, carbapenems and third generation cephalosporins [74]. We also found that isolates showed complete resistance against AMC and AMP but less than 10% resistance rates against all other antibiotic classes tested.

*Escherichia* spp. are known to be involved in a broad spectrum of nosocomial infections, including UTI, septicemia, pneumonia, neonatal meningitis, peritonitis, and gastroenteritis [75]. In our hospital, the genus remained the most isolated (34%) with urine, blood and wound site samples showing the most isolates. Based on the department, most isolates were collected from nephrology, general surgery, gastroenterology, and endocrinology departments. About 15–20% of the isolates showcased ESBL activity with moderate resistance rates against AMC. Resistance against SXT and AMP were relatively higher than AMC. The bacterium showed low resistance against aminoglycosides and carbapenems. Finally, members of the genus *Proteus* spp. are usually involved in community-acquired infections. However, about 10% of the cases are reported to be nosocomial [76]. They are most associated as causative agents of UTI, which usually occurs due to bacterial migration along the mucosal sheath of the catheter or up the catheter lumen from contaminated urine [77,78]. Female sex, immunodeficiency, unprotected sex, longer duration of catheterization, improper catheter cleaning or care etc., are the established risk factors for infection by *Proteus* species. *P. mirabilis* and *P. vulgaris* remain the most isolated species in our hospital. Most of the specimens were isolated from urine and wound site samples with the highest caseload reported by nephrology, neurology, endocrinology, and ICU departments. Resistance against penicillin, amphenicols, quinolones and SXT remained moderate to high, while resistance to carbapenems and third generation cephalosporins remained low. About 25–35% of the samples showed ESBL activity.

### 3.7. AMR Rates in Other Studied Gram-Negative Genera

Of the remaining 10 genera included in the present study, AMR trends were observed for species belonging to four genera—*Aeromonas* spp., *Burkholderia* spp., *Moraxella* spp., and *Stenotrophomonas* spp. Resistance against other genera was not tested, mainly due to low isolation frequency. Aeromonads are emerging nosocomial enteric pathogens that are part of the normal intestinal microflora [79]. They usually colonize the water distribution system by forming biofilms in the water channels, especially during the summer [79,80]. *A. caviae* is the predominant isolate with *A. hydrophila* and *A. veronii* showing specific geographical distribution. Apart from their association with diarrhea (acute, chronic and traveler’s), multiple extra-intestinal manifestations like skin and soft tissue infections, and lower respiratory and urinary tract infections have also been reported [80]. They are universally resistant against penicillin; however, they show susceptibility to aminoglycosides, tetracycline, chloramphenicol, trimethoprim-sulfamethoxazole, and quinolones [79,80,81]. Over four years of the study, only 18 isolates were recorded with nephrology, surgery, and ICU departments with the highest caseload. Our isolates showed less than 10% resistance against CIP and CAZ, while moderate resistance against SXT was noted.

*Burkholderia cepacia* causes severe lung infections in cystic fibrosis and immunocompromised patients. Transmission is usually due to exposure to contaminated solutions such as antiseptics, disinfectants, nebulizer solution, and dextrose solution in a hospitalized patient [82,83,84]. Most of the isolates in the present study were collected from bronchoalveolar lavage, tracheal aspirate, and the wound site. ICU and cardiology departments remained the most burdened. All our isolates were found to be susceptible to CAZ and SXT with no resistance observed. *Moraxella* is a common nosocomial agent in ventilated patients, causing upper and lower respiratory tract infections [85]. Crowding, winter season, prolonged stay in a medical institution, and respiratory therapy are some of the established risk factors for infection [85,86]. Most specimens were collected from sputum and bronchoalveolar lavage in the present study, with thoracic surgery and cardiology having the highest caseload. Resistance was found to be less than 3% against AMC and 0% against amphenicols, quinolones and third-generation cephalosporins.

Finally, *Stenotrophomonas* spp. cause infections that are usually associated with high mortality and morbidity and have been associated with bacteremia, pneumonia, endocarditis, meningitis, UTI, soft tissue and gastrointestinal infections [87]. We isolated most of the specimens from urine and bronchoalveolar lavage samples with surgery, cardiology, and thoracic surgery departments with the highest isolation frequency. Resistance against SXT remained low at roughly 3%, despite the high intrinsic resistance against multiple antimicrobial classes. Amongst the other remaining six genera we included, incidence frequency was high for *Bacteroides* and *Prevotella*. Only a few isolated cases were observed for *Achromobacter, Campylobacter, Chryseobacterium* and *Neisseria*. Mostly, patients with these isolates were treated empirically and were based on relevant national and EU guidelines.

### 3.8. Infection Control and Controlling of AMR Rates

Antimicrobial Stewardship is a crucial and much-needed remedy in controlling AMR rates in nosocomial isolates. It requires a precise and delicate balance involving selection, dosage, and duration of antimicrobial treatment with the end goal being to achieve the best clinical outcome and prevention of infection with minimal impact on subsequent resistance [88]. To achieve the goal, a collaborative effort is required at the hospital level with infectious disease physicians, pharmacists, microbiologists, hospital epidemiologists, and administrative staff joining hands to formulate the necessary strategy [89]. A de-escalating backend approach is seen as the most efficient approach towards achieving stewardship [90,91]. It involves monitoring the current antibiotic prescription patterns and then formulating recommendations for clinicians to either continue, adjust, change, or discontinue the patterns based on all available resources and data [89].

Since this study represents the first of such monitoring efforts on a larger scale, encompassing yearly trends in our hospital, the next logical steps would be to formulate and implement proper strategies to control AMR rates, including antibiotic switching and cycling, dose optimization and prescription controls, in line with EU and ECDC guidelines. Furthermore, we would expand the surveillance and monitoring program to encompass more GNBs apart from ESKAPE and other traditional GNBs. The present study also helps us to determine the baseline levels for caseloads that could, in the future, be used to understand and manage outbreaks in the hospital. In addition to controlling AMR rates, it is equally important to reduce the incidence and caseload of GNBs.

In our hospital, regular meetings and discussions are conducted between laboratory specialists and clinical infectious disease physicians to formulate recommendations regarding the AMR trends and various approaches that can be used in limiting the spread of inter- and intra-departmental nosocomial infections. Our laboratory is available for consultations and offers educational programs including postgraduate training to nurses, physicians, and clinical microbiologists to help them be better prepared to tackle the topical issues of antibiotic misuse and antimicrobial resistance. The obtained data and results from the present study will be used for further training of these specialists, along with the implementation of more stringent and proper infection control methods and hygiene standards.

### 3.9. Limitations of the Present Study

Studies like the present one are essential for the implementation of proper infection control measures, to formulate national prescription guidelines and to control the problem of AMR in HAIs. However, the present study has several limitations. The study did not conduct resistance testing for all antimicrobials against the bacteria isolated, mainly due to limited financial resources. However, testing was done based on the national and EUCAST guidelines and the prescription patterns in the Latvian healthcare settings. We did not perform genotyping to identify specific clonal outbreaks and did not test patients in the outpatient department (community AMR rates). Finally, since this is a single center study, the results may not completely reflect the national AMR rates. However, upon comparison with data available with ECDC, our results agreed with the Latvian national levels as available in ECDC, EARS-Net and ESAC-Net Surveillance reports. In the present study, we did not investigate the prevalence of multidrug-resistant (MDR), extensively drug-resistant (XDR) and pandrug-resistant (PDR) species (due to insufficient testing against all agents according to the guidelines and/or lack of molecular testing). However, according to the WHO guidelines, it is not necessary to define isolates included in the global priority list of AR bacteria as MDR or XDR if they are carbapenem resistant. This is because carbapenemase-encoding genes are carried on mobile genetic elements that usually carry genetic determinants for resistance to other antibiotics [92]. Hence, we just labeled the isolates as carbapenem resistant.

## 4. Materials and Methods

### 4.1. Study Design and Location

A retrospective analysis of antimicrobial susceptibility data from microbiology laboratory was done for the years 2017–2020 (full years from Jan to Dec). The study was conducted at Paul Stradins Clinical University Hospital (PSCUH), Riga, Latvia. PSCUH is a multispecialty hospital providing treatment and care in 26 medical fields catering to patients from both Riga (48%) and other cities and rural areas of Latvia (52%), with an estimated 310,000 patients receiving treatment/consultations each year (as of 2018) [93]. Microbiological data of patients irrespective of age, gender etc., who fulfilled the ECDC (European Center for Disease Prevention and Control) criteria for nosocomial infections, were considered in the present study [94]. Ethical permission for the study was provided by the Clinical Research Ethics Committee of the PSCUH wide no. 290421-16L (29.04.2021).

### 4.2. Sample Collection and Laboratory Analysis

Patient specimens were collected by the clinicians or nurses in respective departments of the hospital and sent for microbiological investigation to the Joint Laboratory. Specimens collected included pus, tracheal swab, blood, urine, catheter, bronchoalveolar lavage etc. Specimens with improper labeling and those with inadequate patient identifiers were excluded from the present study. Furthermore, similar bacterial species isolated from different samples of the same patient were considered as a single isolate. Specimen processing and handling were done in accordance with the relevant EUCAST (European Committee on Antimicrobial Susceptibility Testing) guidelines. Genus and species identification was done using a Vitek2 analyzer or MALDI-TOF MS (matrix assisted laser desorption ionization-time of flight mass spectrometry). Antimicrobial susceptibility for various antibiotics was determined using a standard disk diffusion method in accordance with EUCAST guidelines. A summary of breakpoints used in the present study is shown in Supplementary File 2. EUCAST recommendations on breakpoint for interpretation of zone diameters v11.0 (applicable from Jan 2021) were used for resistance rate analysis [95]. The breakpoints were applied retrospectively to eliminate any variances in interpretation of the results. Isolates identified as Intermediate (I) or Susceptible, Increased exposure (I) were considered as Susceptible (S). The national average antibiotic consumption patterns in the hospital setting were obtained from ESAC-Net (European Surveillance of Antimicrobial Consumption Network) Surveillance reports 2017–2019 and were plotted against the average resistance rates we obtained in the present study.

### 4.3. Multiple Antibiotic Resistance (MAR) Index

By analyzing the resistance rates of a particular bacterial isolate against the total number of tested antimicrobial agents, one can calculate the MAR Index [96]. This tool is an extremely useful source for tracking and analyzing the risk of multi-drug resistance in isolates. The index is calculated as the ratio of number of antimicrobials to which the isolate is resistant, and the total number of antimicrobials exposed to the isolate [96,97]. A MAR Index < 0.2 indicated low risk, while MAR Index ≥ 0.2 indicates a high risk of antimicrobial contamination [96,97].

### 4.4. Data Collection and Analysis

The data about antimicrobial susceptibility, sample type, species, and the department from where the sample was isolated, was downloaded from the laboratory database and analyzed for the period 2017–2020. Differences in antimicrobial susceptibility between the start and the end of the study for each bacterial species was assessed using χ^2^ test. Phi (Φ) was used to determine the significance of positive or negative trends in changes in antimicrobial susceptibility over time. SPSS (v27.0, IBM Corp., Armonk, NY, USA) was used for statistical analysis.

## 5. Conclusions

Antimicrobial resistance (AMR) in gram-negative bacteria (GNBs) poses a significant threat, especially in the healthcare setting. To address the current emerging AMR rates and the increased prevalence of non-traditional GNBs, constant monitoring, along with the revision of antimicrobial guidelines, is needed. The most significant findings of the present study are:

Over the past four years (2017–2020), there have been no significant changes in AMR rates amongst nosocomial isolates of most GNBs. However, the overall resistance rates and the number of GNB isolates in Latvia remain relatively high compared to European averages.

Furthermore, significantly increasing AMR rates in *Klebsiella* spp. and *Enterobacter* spp. isolates need to be handled with care and with proper infection control management.

## Figures and Tables

**Table 1:** Prevalence of different nosocomial gram-negative bacterial genus from 2017–2020.

	Prevalence (%)				Overall Prevalence (%)
	2017	2018	2019	2020	
*Achromobacter* spp.	0.11	0.14	0.10	0.17	00.13
*Acinetobacter* spp.	11.65	9.82	9.72	9.12	10.05
*Aeromonas* spp.	0.11	0.11	0.20	0.20	00.16
*Bacteroides* spp.	0.30	1.39	2.37	3.59	01.95
*Burkholderia* spp.	1.85	0.00	0.60	0.07	00.61
*Campylobacter* spp.	0.00	0.07	0.10	0.10	00.07
*Chryseobacterium* spp.	0.07	0.00	0.07	0.03	00.04
*Citrobacter* spp.	3.07	3.49	3.98	2.70	03.31
*Enterobacter* spp.	7.28	6.90	7.58	8.44	07.56
*Escherichia* spp.	36.78	35.78	34.75	31.74	34.71
*Klebsiella* spp.	16.78	19.84	18.61	21.49	19.22
*Moraxella* spp.	0.18	0.68	0.53	0.48	00.47
*Morganella* spp.	1.37	1.67	1.60	1.64	01.57
*Neisseria* spp.	0.00	0.14	0.17	0.14	00.11
*Prevotella* spp.	0.00	0.96	1.94	2.66	01.43
*Proteus* spp.	6.80	7.43	5.91	5.98	06.51
*Pseudomonas* spp.	6.99	7.82	7.28	7.04	07.28
*Serratia* spp.	1.48	1.85	1.50	1.64	01.62
*Stenotrophomonas* spp.	5.18	1.92	2.97	2.77	03.18
Total	100.00	100.00	100.00	100.00	100.0

## Data Availability

All data analyzed have been presented in a summarized way in Supplementary Files 1 and 2. Patient data cannot be provided due to confidentiality and privacy concerns.

## Supplementary Materials

The following are available online at https://www.mdpi.com/article/10.3390/antibiotics10070791/s1. Supplementary File S1: Microbial Profile and Epidemiology; Supplementary File S2: Antimicrobial Resistance Results; Supplementary File S3: Multiple Antibiotic Resistance (MAR) Index Results.
