# Supplementary material for: Antimicrobial Resistance in Nosocomial Isolates of Gram-Negative Bacteria: Public Health Implications in the Latvian Context
Source: Antibiotics (Basel). 2021 Jun 29;10(7):791. doi: 10.3390/antibiotics10070791 (PMC8300747; doi:10.3390/antibiotics10070791)
Supplement: Supplementary file 1 [file antibiotics-10-00791-s001.zip › Supplementary File S1.pdf]

ADDITIONAL FILE 1
MICROBIAL PROFILE AND EPIDEMIOLOGY

Antimicrobial Resistance in Nosocomial Isolates of Gram-Negative Bacteria: Public Health Implications in Latvian Context

Authors: Nityanand Jain*, Inese Jansone, Tatjana Obidenova, Raimonds Sīmanis, Jānis Meisters, Dagnija Straupmane and Aigars Reinis

*** For Correspondence:**

Department of Biology and Microbiology
Riga Stradins University
16 Dzirciema street
Riga
Latvia (LV-1007)

Email: nityapkl@gmail.com

TABLE OF CONTENTS

1) Distribution of *Achromobacter* species based on clinics
2) Distribution of *Achromobacter* species based on collected sample type
3) Different species of *Achromobacter* isolated over the years

4) Distribution of *Acinetobacter* species based on clinics
5) Distribution of *Acinetobacter* species based on collected sample type
6) Different species of *Acinetobacter* isolated over the years

7) Distribution of *Aeromonas* species based on clinics
8) Distribution of *Aeromonas* species based on collected sample type
9) Different species of *Aeromonas* isolated over the years

10) Distribution of *Bacteroides* species based on clinics
11) Distribution of *Bacteroides* species based on collected sample type
12) Different species of *Bacteroides* isolated over the years

13) Distribution of *Burkholderia* species based on clinics
14) Distribution of *Burkholderia* based on collected sample type
15) Different species of *Burkholderia* isolated over the years

16) Distribution of *Campylobacter* species based on clinics
17) Distribution of *Campylobacter* based on collected sample type
18) Different species of *Campylobacter* isolated over the years

19) Distribution of *Chryseobacterium* species based on clinics
20) Distribution of *Chryseobacterium* based on collected sample type
21) Different species of *Chryseobacterium* isolated over the years

22) Distribution of *Citrobacter* species based on clinics
23) Distribution of *Citrobacter* based on collected sample type
24) Different species of *Citrobacter* isolated over the years

25) Distribution of *Enterobacter* species based on clinics
26) Distribution of *Enterobacter* based on collected sample type
27) Different species of *Enterobacter* isolated over the years

28) Distribution of *Escherichia* species based on clinics

29) Distribution of *Escherichia* species based on collected sample type
30) Different species of *Escherichia* isolated over the years

31) Distribution of *Klebsiella* species based on clinics
32) Distribution of *Klebsiella* based on collected sample type
33) Different species of *Klebsiella* isolated over the years

34) Distribution of *Moraxella* species based on clinics
35) Distribution of *Moraxella* based on collected sample type
36) Different species of *Moraxella* isolated over the years

37) Distribution of *Morganella* species based on clinics
38) Distribution of *Morganella* based on collected sample type
39) Different species of *Morganella* isolated over the years

40) Distribution of *Neisseria* species based on clinics
41) Distribution of *Neisseria* based on collected sample type
42) Different species of *Neisseria* isolated over the years

43) Distribution of *Prevotella* species based on clinics
44) Distribution of *Prevotella* based on collected sample type
45) Different species of *Prevotella* isolated over the years

46) Distribution of *Proteus* species based on clinics
47) Distribution of *Proteus* based on collected sample type
48) Different species of *Proteus* isolated over the years

49) Distribution of *Pseudomonas* species based on clinics
50) Distribution of *Pseudomonas* based on collected sample type
51) Different species of *Pseudomonas* isolated over the years

52) Distribution of *Serratia* species based on clinics
53) Distribution of *Serratia* based on collected sample type
54) Different species of *Serratia* isolated over the years

55) Distribution of *Stenotrophomonas* species based on clinics
56) Distribution of *Stenotrophomonas* based on collected sample type
57) Different species of *Stenotrophomonas* isolated over the years

ACHROMOBACTER

Additional Table 1. Distribution of *Achromobacter* species based on clinics.

Name of Clinic	2017		2018		2019		2020	
	n	%	*n*	%	*n*	%	*n*	%
Intensive Care & Resuscitation	0	0.0	0	0.0	1	33.3	0	0.0
Lung diseases & Thoracic Surgery	2	66.6	0	0.0	2	66.6	1	20.0
Cardiology	0	0.0	1	25.0	0	0.0	0	0.0
Vascular Surgery	0	0.0	0	0.0	0	0.0	1	20.0
Nephrology	0	0.0	2	50.0	0	0.0	0	0.0
Otorhinolaryngology	1	33.3	0	0.0	0	0.0	0	0.0
General Surgery	0	0.0	0	0.0	0	0.0	3	60.0
Endocrinology & Rheumatology	0	0.0	1	25.0	0	0.0	0	0.0
Total Isolates	3 (100%)		4 (100%)		3 (100%)		5 (100%)	

Additional Table 2. Distribution of *Achromobacter* species based on collected sample type.

Sample Type	2017		2018		2019		2020	
	n	%	*n*	%	*n*	%	*n*	%
Bronchoalveolar lavage	2	66.6	0	0.0	2	66.6	0	0.0
Wound	0	0.0	2	50.0	0	0.0	2	40.0
Sputum	1	33.3	0	0.0	1	33.3	1	20.0
Catheter	0	0.0	1	25.0	0	0.0	1	20.0
Trophic ulcer	0	0.0	1	25.0	0	0.0	1	20.0
Total Isolates	3 (100%)		4 (100%)		3 (100%)		5 (100%)	

Additional Table 3. Different species of *Achromobacter* isolated over the years.

Species	2017		2018		2019		2020	
	n	**%**	***n***	**%**	***n***	**%**	***n***	**%**
A. xylosoxidans	3	100.0	4	100.0	3	100.0	5	100.0
Total Isolates	3 (100%)		4 (100%)		3 (100%)		5 (100%)	

ACINETOBACTER

Additional Table 4. Distribution of *Acinetobacter* species based on clinics.

Name of Clinic	2017		2018		2019		2020	
	n	%	*n*	%	*n*	%	*n*	%
Intensive Care & Resuscitation	130	41.3	108	39.1	104	35.7	98	36.7
Lung diseases & Thoracic Surgery	40	12.7	39	14.1	43	14.8	36	13.5
Cardiology (Invasive, Emergency & Surgery)	7	2.2	10	3.6	16	5.5	30	11.2
Cardiac Reanimation	35	11.1	15	5.4	25	8.6	9	3.4
Neonatal & Newborn	8	2.5	3	1.1	3	1.0	4	1.5
Neurology & Neurosurgery	29	9.2	24	8.7	32	11.0	36	13.5
Vascular Surgery	12	3.8	3	1.1	9	3.1	10	3.7
Differential Diagnosis	11	3.5	3	1.1	6	2.1	7	2.6
Nephrology, Urology, Kidney Transplant & Hemodialysis	17	5.4	25	9.1	16	5.5	8	3.0
Otorhinolaryngology	4	1.3	1	0.4	2	0.7	1	0.4
General Surgery	12	3.8	22	8.0	10	3.4	11	4.1
Endocrinology & Rheumatology	5	1.6	11	4.0	11	3.8	7	2.6
Others	5	1.6	10	3.6	14	4.8	10	3.7
Total Isolates	315 (100%)		276 (100%)		291 (100%)		267 (100%)	

Additional Table 5. Distribution of *Acinetobacter* species based on collected sample type.

Sample Type	2017		2018		2019		2020	
	n	%	*n*	%	*n*	%	*n*	%
Bronchoalveolar lavage	75	23.8	74	26.8	135	46.4	108	40.4
Tracheal aspirate	75	23.8	32	11.6	13	4.5	35	13.1
Wound	35	11.1	50	18.1	27	9.3	25	9.4
Urine	22	7.0	36	13.0	23	7.9	23	8.6
Sputum	22	7.0	12	4.3	18	6.2	10	3.7
Blood	15	4.8	20	7.2	16	5.5	6	2.2
Catheter	11	3.5	11	4.0	13	4.5	13	4.9
Trophic ulcer	9	2.9	2	0.7	11	3.8	7	2.6
Abscess	5	1.6	2	0.7	2	0.6	1	0.4
Pus	4	1.3	3	1.1	2	0.6	1	0.4
Pleural fluid	2	0.6	0	0.0	2	0.6	3	1.1
Others	40	12.6	34	12.3	29	9.9	35	13.1
Total Isolates	315 (100%)		276 (100%)		291 (100%)		267 (100%)	

Additional Table 6. Different species of *Acinetobacter* isolated over the years.

Species	2017		2018		2019		2020	
	n	%	*n*	%	*n*	%	*n*	%
A. baumannii	264	83.8	221	80.0	247	84.9	216	80.9
A. johnsonii	1	0.3	4	1.4	2	0.7	3	1.1
A. junii	4	1.3	7	2.5	3	1.0	3	1.1
A. lwoffii	27	8.6	23	8.3	11	3.8	16	5.9
A. pittii	3	0.9	24	8.7	22	7.6	14	5.2
A. calcoaceticus-baumannii complex	13	4.1	1	0.4	1	0.3	0	0.0
Others*	6	1.9	10	3.6	5	1.7	15	5.6
Total Isolates	315 (100%)		276 (100%)		291 (100%)		267 (100%)	

* Other species isolated include:

1) *A. calcoaceticus*
2) *A. haemolyticus*
3) *A. parvus*

AEROMONAS

Additional Table 7. Distribution of *Aeromonas* species based on clinics.

Name of Clinic	2017		2018		2019		2020	
	n	**%**	***n***	**%**	***n***	**%**	***n***	**%**
Intensive Care & Resuscitation	1	33.3	0	0.0	0	0.0	1	16.7
Lung diseases & Thoracic Surgery	0	0.0	1	33.3	0	0.0	0	0.0
Gastroenterology	0	0.0	1	33.3	0	0.0	0	0.0
Vascular Surgery	0	0.0	0	0.0	0	0.0	2	33.3
Differential Diagnosis	0	0.0	0	0.0	0	0.0	1	16.7
Nephrology & Urology	1	33.3	0	0.0	2	33.3	0	0.0
General Surgery	1	33.3	1	33.3	2	33.3	2	33.3
Endocrinology	0	0.0	0	0.0	2	33.3	0	0.0
Total Isolates	3 (100%)		3 (100%)		6 (100%)		6 (100%)	

Additional Table 8. Distribution of *Aeromonas* species based on collected sample type.

Sample Type	2017		2018		2019		2020	
	n	%	*n*	%	*n*	%	*n*	%
Wound	3	100.0	1	33.3	2	33.3	2	33.3
Sputum	0	0.0	0	0.0	0	0.0	1	16.7
Stool	0	0.0	0	0.0	1	16.7	0	0.0
Drain	0	0.0	0	0.0	1	16.7	0	0.0
Blood	0	0.0	1	33.3	0	0.0	0	0.0
Trophic ulcer	0	0.0	0	0.0	0	0.0	2	33.3
Abscess	0	0.0	1	33.3	0	0.0	0	0.0
Others	0	0.0	0	0.0	2	33.3	1	16.7
Total Isolates	3 (100%)		3 (100%)		6 (100%)		6 (100%)	

Additional Table 9. Different species of *Aeromonas* isolated over the years.

Species	2017		2018		2019		2020	
	n	%	*n*	%	*n*	%	*n*	%
A. hydrophila	2	66.6	0	0.0	3	50.0	1	16.7
A. veronii	1	33.3	1	33.3	0	0.0	2	33.3
A. caviae	0	0.0	1	33.3	2	33.3	3	50.0
A. sobria	0	0.0	1	33.3	1	16.7	0	0.0
Total Isolates	3 (100%)		3 (100%)		6 (100%)		6 (100%)	

BACTEROIDES

Additional Table 10. Distribution of *Bacteroides* species based on clinics.

Name of Clinic	2017		2018		2019		2020	
	n	%	*n*	%	*n*	%	*n*	%
Intensive Care & Resuscitation	1	12.5	4	10.3	3	4.2	3	2.9
Lung diseases & Thoracic Surgery	0	0.0	0	0.0	4	5.6	7	6.7
Cardiology (Invasive, Emergency & Surgery)	0	0.0	0	0.0	3	4.2	1	0.9
Oncological Chemotherapy	1	12.5	0	0.0	0	0.0	0	0.0
Oral & Maxillofacial Surgery	0	0.0	0	0.0	1	1.4	8	7.6
Otorhinolaryngology	0	0.0	0	0.0	1	1.4	3	2.9
Neurology	0	0.0	0	0.0	0	0.0	1	0.9
Differential Diagnosis	0	0.0	2	5.1	0	0.0	0	0.0
Nephrology & Urology	0	0.0	6	15.4	6	8.5	1	0.9
Gastroenterology	0	0.0	1	2.6	2	2.8	3	2.9
General Surgery	5	62.5	24	61.5	51	71.8	69	65.7
Endocrinology	1	12.5	2	5.1	0	0.0	7	6.7
Others	0	0.0	0	0.0	0	0.0	2	1.9
Total Isolates	8 (100%)		39 (100%)		71 (100%)		105 (100%)	

Additional Table 11. Distribution of *Bacteroides* species based on collected sample type.

Sample Type	2017		2018		2019		2020	
	n	%	*n*	%	*n*	%	*n*	%
Wound	1	12.5	14	35.9	40	56.3	51	48.6
Blood	4	50.0	2	5.1	1	1.4	2	1.9
Abscess	3	37.5	2	5.1	6	8.4	12	11.4
Pus	0	0.00	2	5.1	3	4.2	1	0.9
Pleural fluid	0	0.00	0	0.00	0	0.00	2	1.9
Others	0	0.00	19	48.7	21	29.6	38	36.2
Total Isolates	8 (100%)		39 (100%)		71 (100%)		105 (100%)	

Additional Table 12. Different species of *Bacteroides* isolated over the years.

Species	2017		2018		2019		2020	
	n	%	*n*	%	*n*	%	*n*	%
B. fragilis	5	62.5	17	43.6	20	28.2	46	43.8
B. ovatus	2	25.0	7	17.9	13	18.3	17	16.2
B. vulgatus	1	12.5	5	12.8	4	5.6	9	8.6
B. caccae	0	0.0	1	2.6	1	1.4	1	0.9
B. thetaiotaomicron	0	0.0	6	15.4	13	18.3	16	15.2
B. uniformis	0	0.0	1	2.6	3	4.2	2	1.9
B. coagulans	0	0.0	0	0.0	1	1.4	1	0.9
B. buccae	0	0.0	0	0.0	0	0.0	1	0.9
Others	0	0.0	2	5.1	16	22.5	12	11.4
Total Isolates	8 (100%)		39 (100%)		71 (100%)		105 (100%)	

BURKHOLDERIA

Additional Table 13. Distribution of *Burkholderia* species based on clinics.

Name of Clinic	2017		2018		2019		2020	
	n	%	*n*	%	*n*	%	*n*	%
Intensive Care & Resuscitation	15	30.0	0	0.0	6	33.3	0	0.0
Lung diseases & Thoracic Surgery	4	8.0	0	0.0	1	5.5	0	0.0
Cardiology (Invasive, Emergency & Surgery)	10	20.0	0	0.0	9	50.0	1	50.0
Cardiac Reanimation	16	32.0	0	0.0	0	0.0	0	0.0
Oncological Chemotherapy	1	2.0	0	0.0	0	0.0	0	0.0
Newborn and Neonatal	0	0.0	0	0.0	1	5.5	0	0.0
Oral & Maxillofacial Surgery	0	0.0	0	0.0	1	5.5	0	0.0
Urology	1	2.0	0	0.0	0	0.0	0	0.0
Otorhinolaryngology	1	2.0	0	0.0	0	0.0	0	0.0
General Surgery	1	2.0	0	0.0	0	0.0	1	50.0
Endocrinology & Rheumatology	1	2.0	0	0.0	0	0.0	0	0.0
Total Isolates	50 (100%)		0 (100%)		18 (100%)		2 (100%)	

Additional Table 14. Distribution of *Burkholderia* species based on collected sample type.

Sample Type	2017		2018		2019		2020	
	n	%	*n*	%	*n*	%	*n*	%
Bronchoalveolar lavage	27	51.9	0	0.0	12	66.6	0	0.0
Tracheal aspirate	12	24.0	0	0.0	2	11.1	0	0.0
Wound	1	2.0	0	0.0	1	5.5	2	100.0
Urine	1	2.0	0	0.0	0	0.0	0	0.0
Sputum	2	4.0	0	0.0	0	0.0	0	0.0
Pus	1	2.0	0	0.0	0	0.0	0	0.0
Pleural fluid	0	0.0	0	0.0	1	5.5	0	0.0
Others	6	12.0	0	0.0	2	11.1	0	0.0
Total Isolates	50 (100%)		0 (100%)		18 (100%)		2 (100%)	

Additional Table 15. Different species of *Burkholderia* isolated over the years.

Species	2017		2018		2019		2020	
	n	%	*n*	%	*n*	%	*n*	%
B. cepacia	50	100.0	0	100.0	18	100.0	2	100.0
Total Isolates	50 (100%)		0 (100%)		18 (100%)		2 (100%)	

CAMPYLOBACTER

Additional Table 16. Distribution of *Campylobacter* species based on clinics.

Name of Clinic	2017		2018		2019		2020	
	n	**%**	***n***	**%**	***n***	**%**	***n***	**%**
Gastroenterology	0	0.0	0	0.0	2	66.6	2	66.6
Differential Diagnosis	0	0.0	0	0.0	1	33.3	1	33.3
Nephrology	0	0.0	1	50.0	0	0.0	0	0.0
Endocrinology	0	0.0	1	50.0	0	0.0	0	0.0
Total Isolates	0 (100%)		2 (100%)		3 (100%)		3 (100%)	

Additional Table 17. Distribution of *Campylobacter* species based on collected sample type.

Sample Type	2017		2018		2019		2020	
	n	**%**	***n***	**%**	***n***	**%**	***n***	**%**
Blood	0	0.0	2	100.0	0	0.0	2	66.6
Stool	0	0.0	0	0.0	3	100.0	1	33.3
Total Isolates	0 (100%)		2 (100%)		3 (100%)		3 (100%)	

Additional Table 18. Different species of *Campylobacter* isolated over the years.

Species	2017		2018		2019		2020	
	n	**%**	***n***	**%**	***n***	**%**	***n***	**%**
C. fetus	0	0.0	1	50.0	0	0.0	0	0.0
C. jejuni	0	0.0	1	50.0	3	100.0	3	100.0
Total Isolates	0 (100%)		2 (100%)		3 (100%)		3 (100%)	

CHRYSEOBACTERIUM

Additional Table 19. Distribution of *Chryseobacterium* species based on clinics.

Name of Clinic	2017		2018		2019		2020	
	n	%	*n*	%	*n*	%	*n*	%
Intensive Care & Resuscitation	1	50.0	0	0.0	1	50.0	0	0.0
Gastroenterology	1	50.0	0	0.0	0	0.0	0	0.0
Cardiology (Invasive, Emergency & Surgery)	0	0.0	0	0.0	1	50.0	0	0.0
Nephrology	0	0.0	0	0.0	0	0.0	1	100.0
Total Isolates	2 (100%)		0 (100%)		2 (100%)		1 (100%)	

Additional Table 20. Distribution of *Chryseobacterium* species based on collected sample type.

Sample Type	2017		2018		2019		2020	
	n	%	*n*	%	*n*	%	*n*	%
Blood	2	100.0	0	0.0	0	0.0	0	0.0
Bronchoalveolar lavage	0	0.0	0	0.0	2	100.0	0	0.0
Wound	0	0.0	0	0.0	0	0.0	1	100.0
Total Isolates	2 (100%)		0 (100%)		2 (100%)		1 (100%)	

Additional Table 21. Different species of *Chryseobacterium* isolated over the years.

Species	2017		2018		2019		2020	
	n	%	*n*	%	*n*	%	*n*	%
C. gleum	1	50.0	0	0.0	1	50.0	0	0.0
C. indologenes	1	50.0	0	0.0	1	50.0	1	100.0
Total Isolates	2 (100%)		0 (100%)		2 (100%)		1 (100%)	

CITROBACTER

Additional Table 22. Distribution of *Citrobacter* species based on clinics.

Name of Clinic	2017		2018		2019		2020	
	n	%	*n*	%	*n*	%	*n*	%
Intensive Care & Resuscitation	2	2.4	9	9.2	8	6.7	2	2.5
Lung diseases & Thoracic Surgery	12	14.5	7	7.1	12	10.1	11	13.9
Cardiology (Invasive, Emergency & Surgery)	6	7.2	13	13.3	17	14.3	9	11.4
Cardiac Reanimation	1	1.2	0	0.0	0	0.0	0	0.0
Neonatal & Newborn	1	1.2	1	1.0	1	0.8	0	0.0
Gastroenterology	6	7.2	11	11.2	9	7.6	3	3.8
Neurology & Neurosurgery	4	4.8	1	1.0	7	5.9	11	13.9
Vascular Surgery	6	7.2	5	5.1	10	8.4	5	6.3
Differential Diagnosis	2	2.4	5	5.1	2	1.7	2	2.5
Nephrology, Urology, Kidney Transplant & Hemodialysis	23	27.7	18	18.4	18	15.1	18	22.8
Otorhinolaryngology	2	2.4	0	0.0	2	1.7	2	2.5
General Surgery	11	13.3	14	14.3	20	16.8	8	10.1
Endocrinology & Rheumatology	6	7.2	10	10.2	6	5.0	5	6.3
Others	1	1.2	4	4.1	7	5.9	3	3.8
Total Isolates	83 (100%)		98 (100%)		119 (100%)		79 (100%)	

Additional Table 23. Distribution of *Citrobacter* species based on collected sample type.

Sample Type	2017		2018		2019		2020	
	n	%	*n*	%	*n*	%	*n*	%
Bronchoalveolar lavage	0	0.0	3	3.1	14	11.8	12	15.2
Tracheal aspirate	9	10.8	1	1.0	0	0.0	0	0.0
Wound	8	9.6	8	8.2	19	16.0	9	11.4
Urine	31	37.3	28	28.6	37	31.1	23	29.1
Sputum	9	10.8	10	10.2	8	6.7	5	6.3
Blood	3	3.6	1	1.0	1	0.8	1	1.3
Catheter	0	0.0	2	2.0	2	1.7	1	1.3
Trophic ulcer	5	6.0	7	7.1	10	8.4	4	5.1
Abscess	5	6.0	5	5.1	4	3.4	0	0.0
Pus	0	0.0	1	1.0	1	0.8	1	1.3
Pleural fluid	0	0.0	1	1.0	1	0.8	0	0.0
Stool	6	7.2	18	18.4	8	6.7	7	8.9
Others	7	8.4	13	13.3	14	11.8	16	20.3
Total Isolates	83 (100%)		98 (100%)		119 (100%)		79 (100%)	

Additional Table 24. Different species of *Citrobacter* isolated over the years.

Species	2017		2018		2019		2020	
	n	%	*n*	%	*n*	%	*n*	%
C. amalonaticus	1	1.2	0	0.0	0	0.0	0	0.0
C. braakii	1	1.2	16	16.3	16	13.4	12	15.2
C. freundii	71	85.5	64	65.3	74	62.2	53	67.1
C. koseri	13	15.7	17	17.3	28	23.5	14	17.7
C. sedlakii	0	0.0	1	1.0	0	0.0	0	0.0
C. farmeri	0	0.0	0	0.0	1	0.8	0	0.0
Total Isolates	83 (100%)		98 (100%)		119 (100%)		79 (100%)	

ENTEROBACTER

Additional Table 25. Distribution of *Enterobacter* species based on clinics.

Name of Clinic	2017		2018		2019		2020	
	n	%	*n*	%	*n*	%	*n*	%
Intensive Care & Resuscitation	32	16.2	19	9.8	21	9.3	36	14.6
Lung diseases & Thoracic Surgery	40	20.3	18	9.3	46	20.3	30	12.1
Cardiology (Invasive, Emergency & Surgery)	25	12.7	17	8.8	39	17.2	44	17.8
Cardiac Reanimation	9	4.6	0	0.0	0	0.0	0	0.0
Neonatal & Newborn	3	1.5	8	4.1	7	3.1	7	2.8
Neurology & Neurosurgery	19	9.6	18	9.3	24	10.6	21	8.5
Vascular Surgery	11	5.6	17	8.8	9	4.0	16	6.5
Differential Diagnosis	3	1.5	7	3.6	6	2.6	3	1.2
Nephrology, Urology, Kidney Transplant & Hemodialysis	32	16.2	54	27.8	32	14.1	34	13.8
Otorhinolaryngology	4	2.0	2	1.0	4	1.8	3	1.2
General Surgery	11	5.6	13	6.7	13	5.7	20	8.1
Endocrinology & Rheumatology	5	2.5	8	4.1	12	5.3	13	5.3
Others	3	1.5	13	6.7	14	6.2	20	8.1
Total Isolates	197 (100%)		194 (100%)		227 (100%)		247 (100%)	

Additional Table 26. Distribution of *Enterobacter* species based on collected sample type.

Sample Type	2017		2018		2019		2020	
	n	%	*n*	%	*n*	%	*n*	%
Bronchoalveolar lavage	38	19.3	27	13.9	67	29.5	65	26.3
Tracheal aspirate	9	4.5	5	2.6	3	1.3	10	4.1
Wound	23	11.7	10	5.2	14	6.2	23	9.3
Urine	47	23.9	62	40.0	51	22.5	50	20.2
Sputum	20	10.2	12	6.2	26	11.5	14	5.7
Blood	9	4.5	13	6.7	8	3.5	11	4.5
Catheter	6	3.0	7	3.6	8	3.5	7	2.8
Trophic ulcer	10	5.1	16	8.2	8	3.5	14	5.7
Abscess	10	5.1	0	0.0	2	0.9	3	1.2
Pus	6	3.0	0	0.0	0	0.0	1	0.4
Pleural fluid	3	1.5	1	0.5	1	0.4	2	0.8
Others	16	8.1	41	21.1	39	17.2	47	19.0
Total Isolates	197 (100%)		194 (100%)		227 (100%)		247 (100%)	

Additional Table 27. Different species of *Enterobacter* isolated over the years.

Species	2017		2018		2019		2020	
	n	%	*n*	%	*n*	%	*n*	%
E. aerogenes	12	6.1	12	6.2	30	13.2	8	3.2
E. asburiae	0	0.0	10	5.1	14	6.2	18	7.4
E. cloacae	185	93.9	172	88.7	182	80.2	214	86.6
E. gergoviae	0	0.0	0	0.0	1	0.4	0	0.0
E. amnigenus	0	0.0	0	0.0	0	0.0	2	0.8
Others	0	0.0	0	0.0	0	0.0	5	2.0
Total Isolates	197 (100%)		194 (100%)		227 (100%)		247 (100%)	

ESCHERICHIA

Additional Table 28. Distribution of *Escherichia* species based on clinics.

Name of Clinic	2017		2018		2019		2020	
	n	%	*n*	%	*n*	%	*n*	%
Intensive Care & Resuscitation	41	4.3	45	4.5	41	4.0	39	4.2
Lung diseases & Thoracic Surgery	84	8.8	42	4.2	63	6.2	46	5.0
Cardiology (Invasive, Emergency & Surgery)	64	6.7	56	5.6	80	7.8	68	7.3
Gastroenterology	35	3.7	60	6.0	72	7.1	105	11.3
Gynecology & Women health	48	5.0	37	3.7	32	3.1	39	4.2
Neonatal & Newborn	66	6.9	62	6.2	53	5.2	42	4.5
Neurology & Neurosurgery	97	10.2	90	8.9	123	12.1	121	13.0
Vascular Surgery	13	1.4	17	1.7	6	0.6	14	1.5
Differential Diagnosis	46	4.8	42	4.2	39	3.8	15	1.6
Nephrology, Urology, Kidney Transplant & Hemodialysis	274	28.7	310	30.8	245	24.0	187	20.1
Otorhinolaryngology	2	0.2	8	0.8	8	0.8	10	1.1
General Surgery	108	11.3	104	10.3	114	11.2	124	13.3
Endocrinology & Rheumatology	67	7.0	116	11.5	152	14.9	94	10.1
Others	10	1.0	17	1.7	12	1.2	25	2.7
Total Isolates	955 (100%)		1006 (100%)		1040 (100%)		929 (100%)	

Additional Table 29. Distribution of *Escherichia* species based on collected sample type.

Sample Type	2017		2018		2019		2020	
	n	%	*n*	%	*n*	%	*n*	%
Bronchoalveolar lavage	21	2.2	33	3.3	70	6.7	55	5.9
Tracheal aspirate	15	1.6	3	0.3	4	0.4	9	1.0
Wound	50	5.3	58	5.8	75	7.2	74	8.0
Urine	622	65.1	649	64.5	669	64.3	538	57.9
Sputum	9	0.9	18	1.8	12	1.2	8	0.9
Blood	68	7.1	67	6.6	78	7.5	76	8.2
Catheter	4	0.4	3	0.3	1	0.1	1	0.1
Trophic ulcer	9	0.9	12	1.2	6	0.6	9	1.0
Abscess	55	5.8	27	2.7	23	2.2	20	2.2
Pus	14	1.5	3	0.3	6	0.6	6	0.6
Pleural fluid	4	0.4	1	0.1	2	0.2	1	0.1
Others	84	8.8	132	13.1	94	9.0	132	14.2
Total Isolates	955 (100%)		1006 (100%)		1040 (100%)		929 (100%)	

Additional Table 30. Different species of *Escherichia* isolated over the years.

Species	2017		2018		2019		2020	
	n	%	*n*	%	*n*	%	*n*	%
E. coli	955	100.0	1004	99.8	1038	99.8	925	99.6
E. hermannii	0	0.0	2	0.2	2	0.2	2	0.2
E. vulneris	0	0.0	0	0.0	0	0.0	2	0.2
Total Isolates	955 (100%)		1006 (100%)		1040 (100%)		929 (100%)	

KLEBSIELLA

Additional Table 31. Distribution of *Klebsiella* species based on clinics.

Name of Clinic	2017		2018		2019		2020	
	n	%	*n*	%	*n*	%	*n*	%
Intensive Care & Resuscitation	81	17.8	90	16.1	69	12.4	88	14.0
Lung diseases & Thoracic Surgery	68	15.0	69	12.4	85	15.3	72	11.4
Cardiology (Invasive, Emergency & Surgery)	32	7.0	74	13.3	75	13.5	81	12.9
Gastroenterology	10	2.2	32	5.7	38	6.8	65	10.3
Oncology & Chemotherapy	4	0.9	0	0.0	2	0.4	9	1.4
Neonatal & Newborn	5	1.1	11	2.0	6	1.1	6	1.0
Neurology & Neurosurgery	55	12.1	41	7.3	52	9.3	65	10.3
Vascular Surgery	9	2.0	9	1.6	14	2.5	11	1.7
Differential Diagnosis	19	4.2	23	4.1	21	3.8	15	2.4
Nephrology, Urology, Kidney Transplant & Hemodialysis	91	20.0	110	19.7	98	17.6	85	13.5
Otorhinolaryngology	11	2.4	13	2.3	14	2.5	14	2.2
General Surgery	35	7.7	51	9.1	50	9.0	64	10.2
Endocrinology & Rheumatology	20	4.4	32	5.7	28	5.0	40	6.4
Others	14	3.1	3	0.5	5	0.9	14	2.2
Total Isolates	454 (100%)		558 (100%)		557 (100%)		629 (100%)	

Additional Table 32. Distribution of *Klebsiella* species based on collected sample type.

**Sample Type**	**2017**		**2018**		**2019**		**2020**	
	n	**%**	***n***	**%**	***n***	**%**	***n***	**%**
Bronchoalveolar lavage	69	15.2	101	18.1	139	24.9	139	22.1
Tracheal aspirate	35	7.7	12	2.2	12	2.2	18	2.9
Wound	29	6.4	42	7.5	59	10.6	52	8.3
Urine	146	32.2	192	34.4	169	30.3	212	33.7
Sputum	22	4.9	38	6.8	39	7.0	25	4.0
Blood	40	8.8	37	6.6	41	7.4	33	5.2
Catheter	8	1.8	6	1.1	5	0.9	5	0.8
Trophic ulcer	9	2.0	9	1.6	17	3.1	9	1.4
Abscess	22	4.8	20	3.6	8	1.4	16	2.5
Pus	12	2.6	8	1.4	4	0.7	3	0.5
Pleural fluid	2	0.4	0	0.0	2	0.4	4	0.6
Others	60	13.2	93	16.7	62	11.1	113	17.9
Total Isolates	454 (100%)		558 (100%)		557 (100%)		629 (100%)	

Additional Table 33. Different species of *Klebsiella* isolated over the years.

**Species**	**2017**		**2018**		**2019**		**2020**	
	n	**%**	***n***	**%**	***n***	**%**	***n***	**%**
K. oxytoca	79	17.4	82	14.7	76	13.6	84	13.4
K. pneumoniae	374	82.4	455	81.5	458	82.2	486	77.3
Others	1	0.2	21	3.7	33	5.9	59	9.3
Total Isolates	454 (100%)		558 (100%)		557 (100%)		629 (100%)	

MORAXELLA

Additional Table 34. Distribution of *Moraxella* species based on clinics.

Name of Clinic	2017		2018		2019		2020	
	n	**%**	***n***	**%**	***n***	**%**	***n***	**%**
Intensive Care & Resuscitation	0	0.0	0	0.0	5	31.3	1	7.1
Lung diseases & Thoracic Surgery	2	40.0	8	42.1	4	25.0	6	42.9
Cardiology (Invasive, Emergency & Surgery)	2	40.0	7	36.7	2	12.5	4	28.6
Gastroenterology	0	0.0	0	0.0	1	6.3	0	0.0
Oral & Maxillofacial Surgery	1	20.0	1	5.3	0	0.0	0	0.0
Ophthalmic Microsurgery	0	0.0	1	5.3	0	0.0	0	0.0
Newborn & Neonatal	0	0.0	0	0.0	0	0.0	2	14.3
Differential Diagnosis	0	0.0	0	0.0	0	0.0	1	7.1
Nephrology	0	0.0	1	5.3	1	6.3	0	0.0
Endocrinology & Rheumatology	0	0.0	0	0.0	3	18.8	0	0.0
Others	0	0.0	1	5.3	0	0.0	0	0.0
Total Isolates	5 (100%)		19 (100%)		16 (100%)		14 (100%)	

Additional Table 35. Distribution of *Moraxella* species based on collected sample type.

Sample Type	2017		2018		2019		2020	
	n	%	*n*	%	*n*	%	*n*	%
Bronchoalveolar lavage	0	0.0	5	26.3	6	37.5	10	71.4
Tracheal aspirate	0	0.0	0	0.0	1	6.3	0	0.0
Sputum	4	80.0	11	57.9	7	43.8	2	14.3
Abscess	1	20.0	0	0.0	0	0.0	0	0.0
Pus	0	0.0	0	0.0	1	6.3	0	0.0
Others	0	0.0	3	15.8	1	6.3	2	14.3
Total Isolates	5 (100%)		19 (100%)		16 (100%)		14 (100%)	

Additional Table 36. Different species of *Moraxella* isolated over the years.

Species	2017		2018		2019		2020	
	n	%	*n*	%	*n*	%	*n*	%
M. catarrhalis	2	40.0	17	89.5	14	87.5	13	92.9
M. osloensis	0	0.0	1	5.2	0	0.0	1	7.1
M. nonliquefaciens	0	0.0	0	0.0	1	6.3	0	0.0
Others	3	60.0	1	5.2	1	6.3	0	0.0
Total Isolates	5 (100%)		19 (100%)		16 (100%)		14 (100%)	

MORGANELLA

Additional Table 37. Distribution of *Morganella* species based on clinics.

Name of Clinic	2017		2018		2019		2020	
	n	%	*n*	%	*n*	%	*n*	%
Intensive Care & Resuscitation	2	5.4	5	10.6	2	4.2	4	8.3
Lung diseases & Thoracic Surgery	4	10.8	4	8.5	4	8.3	3	6.3
Cardiology (Invasive, Emergency & Surgery)	4	10.8	6	12.8	5	10.4	6	12.5
Cardiac Reanimation	2	5.4	0	0.0	0	0.0	0	0.0
Gastroenterology	2	5.4	1	2.1	2	4.2	2	4.2
Gynecology	1	2.7	0	0.0	0	0.0	0	0.0
Neonatal & Newborn	1	2.7	0	0.0	1	2.1	0	0.0
Neurology & Neurosurgery	1	2.7	6	12.8	5	10.4	3	6.3
Vascular Surgery	4	10.8	6	12.8	6	12.5	6	12.5
Differential Diagnosis	1	2.7	2	4.3	1	2.1	2	4.2
Nephrology, Urology, Kidney Transplant & Hemodialysis	5	13.5	7	14.9	12	25.0	12	25.0
Otorhinolaryngology	0	0.0	0	0.0	2	4.2	0	0.0
General Surgery	8	21.6	5	10.6	1	2.1	7	14.6
Endocrinology & Rheumatology	2	5.4	5	10.6	7	14.6	3	6.3
Total Isolates	37 (100%)		47 (100%)		48 (100%)		48 (100%)	

Additional Table 38. Distribution of *Morganella* species based on collected sample type.

Sample Type	2017		2018		2019		2020	
	n	%	*n*	%	*n*	%	*n*	%
Bronchoalveolar lavage	3	8.1	1	2.1	7	14.6	6	12.5
Wound	8	21.6	7	14.9	3	6.3	6	12.5
Urine	10	27.0	23	48.9	22	45.8	12	25.0
Sputum	1	2.7	1	2.1	3	6.3	0	0.0
Blood	1	2.7	1	2.1	2	4.2	3	6.3
Catheter	1	2.7	1	2.1	1	2.1	0	0.0
Trophic ulcer	4	10.8	7	14.9	6	12.5	7	14.6
Abscess	5	13.5	1	2.1	0	0.0	1	2.1
Others	4	10.8	5	10.6	4	8.3	13	27.1
Total Isolates	37 (100%)		47 (100%)		48 (100%)		48 (100%)	

Additional Table 39. Different species of *Morganella* isolated over the years.

Species	2017		2018		2019		2020	
	n	%	*n*	%	*n*	%	*n*	%
M. morganii	37	100.0	47	100.0	48	100.0	48	100.0
Total Isolates	37 (100%)		47 (100%)		48 (100%)		48 (100%)	

NEISSERIA

Additional Table 40. Distribution of *Neisseria* species based on clinics.

Name of Clinic	2017		2018		2019		2020	
	n	%	*n*	%	*n*	%	*n*	%
Intensive Care & Resuscitation	0	0.0	0	0.0	0	0.0	1	25.0
Lung diseases & Thoracic Surgery	0	0.0	0	0.0	2	40.0	1	25.0
Cardiology (Invasive, Emergency & Surgery)	0	0.0	1	25.0	1	20.0	1	25.0
Oral & Maxillofacial Surgery	0	0.0	1	25.0	0	0.0	0	0.0
Ophthalmic Microsurgery	0	0.0	1	25.0	0	0.0	0	0.0
Differential Diagnosis	0	0.0	0	0.0	1	20.0	0	0.0
Nephrology	0	0.0	1	25.0	0	0.0	0	0.0
Otorhinolaryngology	0	0.0	0	0.0	1	20.0	0	0.0
General Surgery	0	0.0	0	0.0	0	0.0	1	25.0
Total Isolates	0 (100%)		4 (100%)		5 (100%)		4 (100%)	

Additional Table 41. Distribution of *Neisseria* species based on collected sample type.

Sample Type	2017		2018		2019		2020	
	n	%	*n*	%	*n*	%	*n*	%
Bronchoalveolar lavage	0	0.0	1	25.0	1	20.0	1	25.0
Wound	0	0.0	0	0.0	1	20.0	1	25.0
Sputum	0	0.0	0	0.0	2	40.0	1	25.0
Others	0	0.0	3	75.0	1	20.0	1	25.0
Total Isolates	0 (100%)		4 (100%)		5 (100%)		4 (100%)	

Additional Table 42. Different species of *Neisseria* isolated over the years.

**Species**	**2017**		**2018**		**2019**		**2020**	
	n	**%**	***n***	**%**	***n***	**%**	***n***	**%**
N. flavescens	0	0.0	1	25.0	1	20.0	1	25.0
N. meningitidis	0	0.0	1	25.0	3	60.0	2	50.0
N. mucosa	0	0.0	1	25.0	0	0.0	0	0.0
N. subflava	0	0.0	1	25.0	0	0.0	1	25.0
Others	0	0.0	0	0.0	1	20.0	0	0.0
Total Isolates	0 (100%)		4 (100%)		5 (100%)		4 (100%)	

PREVOTELLA

Additional Table 43. Distribution of *Prevotella* species based on clinics.

Name of Clinic	2017		2018		2019		2020	
	n	**%**	***n***	**%**	***n***	**%**	***n***	**%**
Intensive Care & Resuscitation	0	0.0	1	3.7	3	5.2	2	2.5
Lung diseases & Thoracic Surgery	0	0.0	4	14.9	3	5.2	6	7.7
Cardiology (Invasive, Emergency & Surgery)	0	0.0	0	0.0	1	1.7	0	0.0
Gastroenterology	0	0.0	0	0.0	0	0.0	1	1.3
Oral & Maxillofacial Surgery	0	0.0	13	48.1	27	46.6	37	47.5
Nephrology	0	0.0	1	3.7	0	0.0	0	0.0
Otorhinolaryngology	0	0.0	3	11.1	6	10.3	11	14.2
General Surgery	0	0.0	2	7.4	17	29.3	15	19.2
Endocrinology & Rheumatology	0	0.0	3	11.1	1	1.7	3	3.8
Others	0	0.0	0	0.0	0	0.0	3	3.8
Total Isolates	0 (100%)		27 (100%)		58 (100%)		78 (100%)	

Additional Table 44. Distribution of *Prevotella* species based on collected sample type.

Sample Type	2017		2018		2019		2020	
	n	%	*n*	%	*n*	%	*n*	%
Wound	0	0.0	2	7.4	14	24.1	12	15.4
Blood	0	0.0	1	3.7	1	1.7	0	0.0
Abscess	0	0.0	4	14.8	16	27.6	17	21.8
Pus	0	0.0	1	3.7	4	6.9	2	2.6
Pleural fluid	0	0.0	2	7.4	0	0.0	2	2.6
Others	0	0.0	17	62.9	23	39.7	45	57.7
Total Isolates	0 (100%)		27 (100%)		58 (100%)		78 (100%)	

Additional Table 45. Different species of *Prevotella* isolated over the years.

Species	2017		2018		2019		2020	
	n	%	*n*	%	*n*	%	*n*	%
P. bivia	0	0.0	2	7.4	3	5.2	0	0.0
P. buccae	0	0.0	3	11.1	13	22.4	14	17.9
P. denticola	0	0.0	3	11.1	4	6.9	4	5.1
P. disiens	0	0.0	1	3.7	2	3.4	1	1.3
P. heparinolytica	0	0.0	2	7.4	1	1.7	3	3.8
P. intermedia	0	0.0	6	22.2	9	15.5	10	12.8
P. melaninogenica	0	0.0	1	3.7	9	15.5	8	10.3
P. nigrescens	0	0.0	3	11.1	7	12.1	5	6.4
P. oris	0	0.0	3	11.1	1	1.7	1	1.3
Others	0	0.0	3	11.1	9	15.5	32	41.0
Total Isolates	0 (100%)		27 (100%)		58 (100%)		76 (100%)	

PROTEUS

Additional Table 46. Distribution of *Proteus* species based on clinics.

Name of Clinic	2017		2018		2019		2020	
	n	**%**	***n***	**%**	***n***	**%**	***n***	**%**
Intensive Care & Resuscitation	23	12.5	17	8.1	17	9.6	21	12.0
Lung diseases & Thoracic Surgery	24	13.0	16	7.7	13	7.3	12	6.9
Cardiology (Invasive, Emergency & Surgery)	13	7.1	16	7.7	19	10.7	14	8.0
Gastroenterology	6	3.3	13	6.2	6	3.4	8	4.6
Neurology & Neurosurgery	20	10.9	22	10.5	23	13.0	18	10.3
Vascular Surgery	9	4.9	19	9.1	17	9.6	13	7.4
Differential Diagnosis	8	4.3	19	9.1	13	7.3	16	9.1
Nephrology, Urology, Kidney Transplant & Hemodialysis	45	24.5	50	23.9	35	19.8	31	17.7
Otorhinolaryngology	2	1.1	3	1.4	5	2.8	1	0.6
General Surgery	19	10.3	17	8.1	10	5.6	16	9.1
Endocrinology & Rheumatology	11	6.0	13	6.2	18	10.2	18	10.3
Others	4	2.2	4	1.9	1	0.6	7	4.0
Total Isolates	184 (100%)		209 (100%)		177 (100%)		175 (100%)	

Additional Table 47. Distribution of *Proteus* species based on collected sample type.

Sample Type	2017		2018		2019		2020	
	n	%	*n*	%	*n*	%	*n*	%
Bronchoalveolar lavage	12	6.5	8	3.8	23	13.0	20	11.4
Tracheal aspirate	7	3.8	4	1.9	6	3.4	7	4.0
Wound	26	14.1	32	15.3	20	11.3	27	15.4
Urine	77	41.8	94	45.0	61	34.5	63	36.0
Sputum	1	0.5	5	2.4	2	1.1	0	0.0
Blood	11	6.0	9	4.3	12	6.8	5	2.9
Catheter	3	1.6	4	1.9	4	2.3	1	0.6
Trophic ulcer	7	3.8	19	9.1	19	10.7	14	8.0
Abscess	10	5.4	2	1.0	1	0.6	3	1.7
Pus	5	2.7	1	0.5	1	0.6	2	1.1
Pleural fluid	0	0.0	0	0.0	0	0.0	2	1.1
Others	25	13.6	31	14.8	28	15.8	31	17.7
Total Isolates	184 (100%)		209 (100%)		177 (100%)		175 (100%)	

Additional Table 48. Different species of *Proteus* isolated over the years.

Species	2017		2018		2019		2020	
	n	%	*n*	%	*n*	%	*n*	%
P. mirabilis	144	78.3	179	85.6	152	85.9	149	85.1
P. penneri	1	0.5	2	1.0	0	0.0	0	0.0
P. vulgaris	39	21.2	22	10.5	16	9.0	18	10.3
Others	0	0.0	6	2.9	9	5.1	8	4.6
Total Isolates	184 (100%)		209 (100%)		177 (100%)		175 (100%)	

PSEUDOMONAS

Additional Table 49. Distribution of *Pseudomonas* species based on clinics.

Name of Clinic	2017		2018		2019		2020	
	n	%	*n*	%	*n*	%	*n*	%
Intensive Care & Resuscitation	21	11.1	25	11.4	25	11.5	42	20.4
Lung diseases & Thoracic Surgery	36	19.0	30	13.6	35	16.1	24	11.7
Cardiology (Invasive, Emergency & Surgery)	25	13.2	24	10.9	18	8.3	27	13.1
Gastroenterology	8	4.2	9	4.1	9	4.1	5	2.4
Neurology & Neurosurgery	15	7.9	19	8.6	22	10.1	10	4.9
Vascular Surgery	13	6.9	9	4.1	7	3.2	16	7.8
Differential Diagnosis	10	5.3	9	4.1	13	6.0	8	3.9
Nephrology, Urology, Kidney Transplant & Hemodialysis	42	22.2	48	21.8	38	17.4	34	16.5
Otorhinolaryngology	5	2.6	10	4.5	6	2.8	4	1.9
General Surgery	11	5.8	12	5.5	21	9.6	18	8.7
Endocrinology & Rheumatology	3	1.6	13	5.9	21	9.6	15	7.3
Others	0	0.0	12	5.5	3	1.4	3	1.5
Total Isolates	189 (100%)		220 (100%)		218 (100%)		206 (100%)	

Additional Table 50. Distribution of *Pseudomonas* species based on collected sample type.

Sample Type	2017		2018		2019		2020	
	n	%	*n*	%	*n*	%	*n*	%
Bronchoalveolar lavage	29	15.3	37	16.8	51	23.4	45	21.8
Tracheal aspirate	11	5.8	4	1.8	4	1.8	13	6.3
Wound	25	13.2	33	15.0	26	11.9	24	11.7
Urine	60	31.7	60	27.3	65	29.8	51	24.8
Sputum	15	8.0	19	8.6	24	11.0	10	4.9
Blood	7	3.7	7	3.2	11	5.0	5	2.4
Catheter	2	1.1	4	1.8	4	1.8	4	1.9
Trophic ulcer	12	6.3	9	4.1	7	3.2	18	8.7
Abscess	5	2.6	3	1.4	0	0.0	4	1.9
Pus	6	3.2	3	1.4	2	0.9	0	0.0
Pleural fluid	0	0.0	0	0.0	1	0.5	1	0.5
Others	17	9.0	41	18.6	23	10.6	31	15.0
Total Isolates	189 (100%)		220 (100%)		218 (100%)		206 (100%)	

Additional Table 51. Different species of *Pseudomonas* isolated over the years.

Species	2017		2018		2019		2020	
	n	%	*n*	%	*n*	%	*n*	%
P. aeruginosa	162	85.7	192	87.3	197	90.4	185	89.8
P. fluorescens	6	3.2	5	2.3	0	0.0	3	1.5
P. putida	13	6.8	8	3.6	6	2.8	11	5.3
P. stutzeri	2	1.1	4	1.8	4	1.8	2	1.0
Others*	6	3.2	11	5.0	11	5.0	5	2.4
Total Isolates	189 (100%)		220 (100%)		218 (100%)		206 (100%)	

*Other species isolated include:

1) *P. alcaligenes*
2) *P. luteola*
3) *P. oryzihabitans*
4) *P. mendocina*

SERRATIA

Additional Table 52. Distribution of *Serratia* species based on clinics.

Name of Clinic	2017		2018		2019		2020	
	n	**%**	**n**	**%**	***n***	**%**	***n***	**%**
Intensive Care & Resuscitation	3	7.5	7	13.5	4	8.9	8	16.7
Lung diseases & Thoracic Surgery	7	17.5	14	26.9	10	22.2	12	25.0
Cardiology (Invasive, Emergency & Surgery)	9	22.5	11	21.2	5	11.1	6	12.5
Gastroenterology	1	2.5	1	1.9	4	8.9	0	0.0
General Surgery	0	0.0	4	7.7	5	11.1	1	2.1
Neurology & Neurosurgery	5	12.5	2	3.8	4	8.9	2	4.2
Vascular Surgery	4	10.0	3	5.8	3	6.7	5	10.4
Differential Diagnosis	0	0.0	1	1.9	1	2.2	3	6.3
Nephrology, Urology, Kidney Transplant & Hemodialysis	4	10.0	2	3.8	4	8.9	8	16.7
Neonatal & Newborn	0	0.0	1	1.9	2	4.4	1	2.1
Otorhinolaryngology	0	0.0	2	3.8	2	4.4	0	0.0
Endocrinology & Rheumatology	1	2.5	2	3.8	1	2.2	1	2.1
Others	6	15.0	2	3.8	0	0.0	1	2.1
Total Isolates	40 (100%)		52 (100%)		45 (100%)		48 (100%)	

Additional Table 53. Distribution of *Serratia* species based on collected sample type.

Sample Type	2017		2018		2019		2020	
	n	%	*n*	%	*n*	%	*n*	%
Bronchoalveolar lavage	11	27.5	13	25.0	12	26.7	10	20.8
Tracheal aspirate	2	5.0	1	1.9	3	6.7	10	20.8
Wound	6	15.0	6	11.5	10	22.2	5	10.4
Urine	6	15.0	6	11.5	6	13.3	8	16.7
Sputum	4	10.0	7	13.5	4	8.9	8	16.7
Blood	2	5.0	4	7.7	3	6.7	2	4.2
Catheter	0	0.0	3	5.8	0	0.0	1	2.1
Trophic ulcer	4	10.0	3	5.8	3	6.7	4	8.3
Abscess	1	2.5	2	3.8	1	2.2	0	0.0
Pus	0	0.0	0	0.0	0	0.0	0	0.0
Pleural fluid	0	0.0	0	0.0	0	0.0	0	0.0
Others	4	10.0	7	13.5	3	6.7	0	0.0
Total Isolates	40 (100%)		52 (100%)		45 (100%)		48 (100%)	

Additional Table 54. Different species of *Serratia* isolated over the years.

Species	2017		2018		2019		2020	
	n	%	*n*	%	*n*	%	*n*	%
S. fonticola	2	5.0	0	0.0	0	0.0	0	0.0
S. liquefaciens	2	5.0	7	13.5	5	11.1	12	25.0
S. marcescens	35	87.5	45	86.5	39	86.7	33	68.7
S. odorifera	1	2.5	0	0.0	0	0.0	1	2.1
S. plymuthica	0	0.0	0	0.0	1	2.2	1	2.1
S. rubidaea	0	0.0	0	0.0	0	0.0	1	2.1
Total Isolates	40 (100%)		52 (100%)		45 (100%)		48 (100%)	

STENOTROPHOMONAS

Additional Table 55. Distribution of *Stenotrophomonas* species based on clinics.

Name of Clinic	2017		2018		2019		2020	
	n	%	*n*	%	*n*	%	*n*	%
Intensive Care & Resuscitation	18	12.9	6	11.1	10	11.2	21	25.9
Lung diseases & Thoracic Surgery	33	23.6	10	18.5	15	16.9	12	14.8
Cardiology (Invasive, Emergency & Surgery)	7	5.0	10	18.5	20	22.5	12	14.8
Gastroenterology	6	4.3	2	3.7	7	7.9	4	4.9
General Surgery	0	0.0	1	1.9	2	2.2	4	4.9
Neurology & Neurosurgery	12	8.6	3	5.6	3	3.4	6	7.4
Vascular Surgery	15	10.7	3	5.6	12	13.5	7	8.6
Differential Diagnosis	0	0.0	5	9.3	2	2.2	1	1.2
Nephrology, Urology, Kidney Transplant & Hemodialysis	36	25.7	8	14.8	11	12.4	7	8.6
Otorhinolaryngology	0	0.0	1	1.9	1	1.1	2	2.5
Endocrinology & Rheumatology	6	4.3	1	1.9	4	4.5	5	6.2
Others	7	5.0	4	7.4	2	2.2	0	0.0
Total Isolates	140 (100%)		54 (100%)		89 (100%)		81 (100%)	

Additional Table 56. Distribution of *Stenotrophomonas* species based on collected sample type.

Sample Type	2017		2018		2019		2020	
	n	%	*n*	%	*n*	%	*n*	%
Bronchoalveolar lavage	28	20.0	17	31.5	33	37.1	29	35.8
Tracheal aspirate	3	2.1	3	5.5	2	2.2	8	9.9
Wound	21	15.0	4	7.4	7	7.9	3	3.7
Urine	42	30.0	13	24.1	7	7.9	7	8.6
Sputum	21	15.0	4	7.4	11	12.4	5	6.2
Blood	6	4.3	1	1.9	4	4.5	9	11.1
Catheter	3	2.1	1	1.9	1	1.1	2	2.5
Trophic ulcer	12	8.6	3	5.5	12	13.5	9	11.1
Abscess	0	0.0	2	3.7	1	1.1	1	1.2
Pus	0	0.0	1	1.9	1	1.1	0	0.0
Pleural fluid	0	0.0	1	1.9	0	0.0	1	1.2
Others	4	2.9	4	7.4	10	11.2	7	8.6
Total Isolates	140 (100%)		54 (100%)		89 (100%)		81 (100%)	

Additional Table 57. Different species of *Stenotrophomonas* isolated over the years.

Species	2017		2018		2019		2020	
	n	%	*n*	%	*n*	%	*n*	%
S. maltophilia	140	100.0	54	100.0	87	97.8	80	98.8
Others	0	0.0	0	0.0	2	2.1	1	1.2
Total Isolates	140 (100%)		54 (100%)		89 (100%)		81 (100%)	
