# Supplementary material for: Antimicrobial Resistance in Nosocomial Isolates of Gram-Negative Bacteria: Public Health Implications in the Latvian Context
Source: Antibiotics (Basel). 2021 Jun 29;10(7):791. doi: 10.3390/antibiotics10070791 (PMC8300747; doi:10.3390/antibiotics10070791)
Supplement: Supplementary file 1 [file antibiotics-10-00791-s001.zip › Supplementary File S2.pdf]

<u>ADDITIONAL FILE 2</u>
ANTIMICROBIAL RESISTANCE RESULTS

Antimicrobial Resistance in Nosocomial Isolates of Gram-Negative Bacteria: Public Health Implications in Latvian Context

Authors: Nityanand Jain*, Inese Jansone, Tatjana Obidenova, Raimonds Sīmanis, Jānis Meisters, Dagnija Straupmane and Aigars Reinis

Reference values as adapted from

"The European Committee on Antimicrobial Susceptibility Testing (EUCAST). Breakpoint tables for interpretation of MICs and zone diameters. v11.0, 2021. http://www.eucast.org"

*** For Correspondence:**

Department of Biology and Microbiology
Riga Stradins University
16 Dzirciema street
Riga
Latvia (LV-1007)

Email: nityapkl@gmail.com

INDEX

1) Additional Tables 1 & 2 – Antimicrobial resistance rates in *Acinetobacter* species
2) Additional Tables 3 & 4 – Antimicrobial resistance rates in *Aeromonas* species
3) Additional Tables 5 & 6 – Antimicrobial resistance rates in *Burkholderia* species
4) Additional Tables 7 & 8 – Antimicrobial resistance rates in *Citrobacter* species
5) Additional Tables 9 & 10 – Antimicrobial resistance rates in *Enterobacter* species
6) Additional Tables 11 & 12 – Antimicrobial resistance rates in *Escherichia* species
7) Additional Tables 13 & 14 – Antimicrobial resistance rates in *Klebsiella* species
8) Additional Tables 15 & 16 – Antimicrobial resistance rates in *Moraxella* species
9) Additional Tables 17 & 18 – Antimicrobial resistance rates in *Morganella* species
10) Additional Tables 19 & 20 – Antimicrobial resistance rates in *Proteus* species
11) Additional Tables 21 & 22 – Antimicrobial resistance rates in *Pseudomonas* species
12) Additional Tables 23 & 24 – Antimicrobial resistance rates in *Serratia* species
13) Additional Tables 25 & 26 – Antimicrobial resistance rates in *Stenotrophomonas* species

* Antimicrobial resistance data is not available for six genera – *Achromobacter, Bacteroides, Campylobacter, Chryseobacterium, Neisseria and Prevotella*.

ACINETOBACTER

Additional Table 1. Antimicrobial resistance (R) in *Acinetobacter* species from 2017-2020

Antimicrobial Agent	Antimicrobial Resistance % (R)				Overall Change (2017 vs 2020)
	2017	2018	2019	2020	
Amikacin (AMK)	90.67 %	55.21 %	67.36 %	65.68 %	↓
Colistin (COL)	00.00 %	00.00 %	00.00 %	00.00 %	=
Gentamicin (GEN)	71.09 %	56.29 %	58.89 %	57.78 %	↓
Imipenem (IPM)	75.00 %	66.09 %	74.65 %	70.00 %	↓
Trimethoprim-sulfamethoxazole (SXT)	74.25 %	63.60 %	49.47 %	30.99%	↓

* NT – not tested; ↑ indicates increase in resistance; ↓ indicates decrease in resistance; = indicates no change in resistance

Additional Table 2. Breakpoints for antibiotics against *Acinetobacter* species in disk diffusion method (EUCAST guidelines v11.0; applicable Jan 2021)

Antibiotic	Disk Content (μg)	Susceptible (S) [in mm]		Resistant (R) [in mm]	Notes
		Susceptible, standard dosing (S)	Susceptible, increased exposure (I)		
AMK	30	≥ 19	-	< 19	
COL	-	≤ 2	-	> 2	Broth microdilution was performed. The values indicate MIC (minimum inhibitory concentration) cut-offs.
GEN	10	≥ 17	-	< 17	
IPM	10	≥ 24	21 – 23	< 21	
SXT	1.25 – 23.75	≥ 14	11 – 13	< 11	

AEROMONAS

Additional Table 3. Antimicrobial resistance (R) in *Aeromonas* species from 2017-2020

Antimicrobial Agent	Antimicrobial Resistance % (R)				Overall Change (2018 vs 2020)
	2017	2018	2019	2020	
Ceftazidime (CAZ)	NT	0.00 %	0.00 %	25.00 %	↑
Ciprofloxacin (CIP)	NT	0.00 %	25.00 %	0.00 %	=
Trimethoprim-sulfamethoxazole (SXT)	NT	33.33 %	75.00 %	0.00 %	↓

* NT – not tested; ↑ indicates increase in resistance; ↓ indicates decrease in resistance; = indicates no change in resistance

Additional Table 4. Breakpoints for antibiotics against *Aeromonas* species in disk diffusion method (EUCAST guidelines v11.0; applicable Jan 2021)

Antibiotic	Disk Content (µg)	Susceptible (S) [in mm]		Resistant (R) [in mm]	Notes
		Susceptible, standard dosing (S)	Susceptible, increased exposure (I)		
CAZ	10	≥ 24	21 – 23	< 21	
CIP	5	≥ 27	24 – 26	< 24	
SXT	1.25 – 23.75	≥ 19	16 – 18	< 16	

BURKHOLDERIA

Additional Table 5. Antimicrobial resistance (R) in *Burkholderia* species from 2017-2020

Antimicrobial Agent	Antimicrobial Resistance % (R)				Overall Change (2017 vs 2020)
	2017	2018	2019	2020	
Ceftazidime (CAZ)	0.00 %	NT	0.00 %	0.00 %	=
Meropenem (MEM)	0.00 %	NT	5.55 %	0.00 %	=
Minocycline (MNO)	0.00 %	NT	0.00 %	0.00 %	=
Trimethoprim-sulfamethoxazole (SXT)	0.00 %	NT	0.00 %	0.00 %	=

* NT – not tested; ↑ indicates increase in resistance; ↓ indicates decrease in resistance; = indicates no change in resistance

Additional Table 6. Breakpoints for antibiotics against *Burkholderia* species in disk diffusion method (EUCAST guidelines v11.0; applicable Jan 2021)

According to EUCAST v11.0 guidelines, breakpoints for Burkholderia cepacia complex organisms are not determined since accurate and reproducible methods for antimicrobial susceptibility testing are lacking due to technical difficulties encountered with these species and the lack of convincing clinical outcome correlates.

Antibiotic	Disk Content (μg)	Susceptible (S) [in mm]		Resistant (R) [in mm]	Notes
		Susceptible, standard dosing (S)	Susceptible, increased exposure (I)		
CAZ	30	-	-	-	
MEM	10	-	-	-	
MNO	30	-	-	-	
SXT	1.25 – 23.75	-	-	-	

CITROBACTER

Additional Table 7. Antimicrobial resistance (R) in *Citrobacter* species from 2017-2020

Antimicrobial Agent	Antimicrobial Resistance % (R)				Overall Change (2017 vs 2020)
	2017	2018	2019	2020	
Amoxicillin-clavulanic acid (AMC)	80.00 %	83.67 %	75.95 %	85.71 %	↑
AMC_UTI	82.61 %	72.00 %	75.00 %	80.95 %	↓
Amikacin (AMK)	01.59 %	00.00 %	00.00 %	00.00 %	↓
Ampicillin (AMP)	100.00 %	100.00 %	100.00 %	98.57 %	↓
Ceftazidime (CAZ)	26.56 %	23.61 %	26.79 %	28.57 %	↑
Chloramphenicol (CHL)	07.32 %	08.51 %	16.22 %	04.00 %	↓
Ciprofloxacin (CIP)	17.20 %	17.57 %	15.19 %	11.27 %	↓
Cefotaxime (CTX)	25.00 %	26.03 %	27.68 %	25.71 %	↑
Ertapenem (ETP)	04.62 %	09.46 %	09.09 %	09.86 %	↑
Gentamicin (GEN)	15.63 %	12.16 %	10.71 %	09.72 %	↓
Imipenem (IPM)	01.56 %	01.33 %	00.89 %	00.00 %	↓
Meropenem (MEM)	01.56 %	00.00 %	00.00 %	00.00 %	↓
Trimethoprim-sulfamethoxazole (SXT)	20.31 %	12.33 %	14.68 %	15.49 %	↓
Piperacillin-tazobactam (TZP)	21.54 %	14.86 %	17.69 %	09.72 %	↓

* NT – not tested; UTI – uncomplicated urinary tract infection; ↑ indicates increase in resistance; ↓ indicates decrease in resistance; = indicates no change in resistance

Additional Table 8. Breakpoints for antibiotics against *Citrobacter* species in disk diffusion method (EUCAST guidelines v11.0; applicable Jan 2021)

Antibiotic	Disk Content (µg)	Susceptible (S) [in mm]		Resistant (R) [in mm]	Notes
		Susceptible, standard dosing (S)	Susceptible, increased exposure (I)		
AMC	10 – 20	≥ 19	-	< 19	
AMC_UTI	10 – 20	≥ 16	-	< 16	
AMK	30	≥ 18	-	< 18	
AMP	10	≥ 14	-	< 14	
CAZ	10	≥ 22	19 – 21	< 19	
CHL	30	≥ 17	-	< 17	
CIP	5	≥ 25	22 – 24**	< 22	** ATU – area of technical uncertainty
CTX	5	≥ 20	17 – 19	< 17	
ETP	10	≥ 25	-	< 25	
GEN	10	≥ 17	-	< 17	
IPM	10	≥ 22	19 – 21	< 19	
MEM	10	≥ 22	16 – 21	< 16	
SXT	1.25 -23.75	≥ 14	11 – 13	< 11	
TZP	30 – 6	≥ 20	19**	< 20	** ATU – area of technical uncertainty

ENTEROBACTER

Additional Table 9. Antimicrobial resistance (R) in *Enterobacter* species from 2017-2020

Antimicrobial Agent	Antimicrobial Resistance % (R)				Overall Change (2017 vs 2020)
	2017	2018	2019	2020	
Amoxicillin-clavulanic acid (AMC)	100.00 %	100.00 %	100.00 %	100.00 %	=
AMC_UTI	100.00 %	100.00 %	100.00 %	100.00 %	=
Amikacin (AMK)	00.57 %	00.00 %	00.00 %	00.00 %	↓
Ampicillin (AMP)	99.42 %	100.00 %	100.00 %	100.00 %	↑
Ceftazidime (CAZ)	42.77 %	37.23 %	34.93 %	40.16 %	↓
Chloramphenicol (CHL)	28.03 %	23.02 %	15.61 %	14.06 %	↓
Ciprofloxacin (CIP)	29.89 %	19.46 %	19.30 %	15.57 %	↓
Cefotaxime (CTX)	43.35 %	38.62 %	34.96 %	41.56 %	↓
Ertapenem (ETP)	06.29 %	20.53 %	16.44 %	24.70 %	↑
Gentamicin (GEN)	32.76 %	20.11 %	17.62 %	10.70 %	↓
Imipenem (IPM)	01.15 %	01.58 %	00.43 %	00.00 %	↓
Meropenem (MEM)	00.57 %	01.05 %	00.00 %	00.00 %	↓
Trimethoprim-sulfamethoxazole (SXT)	34.10 %	22.87 %	21.78 %	16.73 %	↓
Piperacillin-tazobactam (TZP)	23.43 %	25.00 %	24.45 %	24.79 %	↑

* NT – not tested; UTI – uncomplicated urinary tract infection; ↑ indicates increase in resistance; ↓ indicates decrease in resistance; = indicates no change in resistance

Additional Table 10. Breakpoints for antibiotics against *Enterobacter* species in disk diffusion method (EUCAST guidelines v11.0; applicable Jan 2021)

Antibiotic	Disk Content (μg)	Susceptible (S) [in mm]		Resistant (R) [in mm]	Notes
		Susceptible, standard dosing (S)	Susceptible, increased exposure (I)		
AMC	10 – 20	≥ 19	-	< 19	
AMC_UTI	10 – 20	≥ 16	-	< 16	
AMK	30	≥ 18	-	< 18	
AMP	10	≥ 14		< 14	
CAZ	10	≥ 22	19 – 21	< 19	
CHL	30	≥ 17	-	< 17	
CIP	5	≥ 25	22 – 24**	< 22	** ATU – area of technical uncertainty
CTX	5	≥ 20	17 – 19	< 17	
ETP	10	≥ 25	-	< 25	
GEN	10	≥ 17	-	< 17	
IPM	10	≥ 22	19 – 21	< 19	
MEM	10	≥ 22	16 – 21	< 16	
SXT	1.25 -23.75	≥ 14	11 – 13	< 11	
TZP	30 – 6	≥ 20	19**	< 20	** ATU – area of technical uncertainty

ESCHERICHIA

Additional Table 11. Antimicrobial resistance (R) in *Escherichia* species from 2017-2020

Antimicrobial Agent	Antimicrobial Resistance % (R)				Overall Change (2017 vs 2020)
	2017	2018	2019	2020	
Amoxicillin-clavulanic acid (AMC)	24.52 %	27.37 %	33.22 %	31.10 %	↑
AMC_UTI	13.53 %	12.04 %	13.58 %	14.80 %	↑
Amikacin (AMK)	00.43 %	00.63 %	00.66 %	00.65 %	↑
Ampicillin (AMP)	51.55 %	55.63 %	55.96 %	53.75 %	↑
Ceftazidime (CAZ)	13.67 %	12.40 %	12.60 %	14.23 %	↑
Chloramphenicol (CHL)	07.66 %	10.75 %	10.67 %	09.01 %	↑
Ciprofloxacin (CIP)	25.18 %	24.60 %	21.46 %	22.95 %	↓
Cefotaxime (CTX)	15.97 %	15.76 %	15.07 %	18.50 %	↑
Ertapenem (ETP)	00.14 %	00.31 %	00.13 %	00.52 %	↑
Gentamicin (GEN)	10.81 %	07.15 %	06.55 %	10.16 %	↓
Imipenem (IPM)	00.00 %	00.15 %	00.00 %	00.00 %	=
Meropenem (MEM)	00.00 %	00.00 %	00.00 %	00.00 %	=
Trimethoprim-sulfamethoxazole (SXT)	30.00 %	33.07 %	29.66 %	29.34 %	↓
Piperacillin-tazobactam (TZP)	05.16 %	03.00 %	02.93 %	02.19 %	↓

* NT – not tested; UTI – uncomplicated urinary tract infection; ↑ indicates increase in resistance; ↓ indicates decrease in resistance; = indicates no change in resistance

Additional Table 12. Breakpoints for antibiotics against *Escherichia* species in disk diffusion method (EUCAST guidelines v11.0; applicable Jan 2021)

Antibiotic	Disk Content (µg)	Susceptible (S) [in mm]		Resistant (R) [in mm]	Notes
		Susceptible, standard dosing (S)	Susceptible, increased exposure (I)		
AMC	10 – 20	≥ 19	-	< 19	
AMC_UTI	10 – 20	≥ 16	-	< 16	
AMK	30	≥ 18	-	< 18	
AMP	10	≥ 14		< 14	
CAZ	10	≥ 22	19 – 21	< 19	
CHL	30	≥ 17	-	< 17	
CIP	5	≥ 25	22 – 24**	< 22	** ATU – area of technical uncertainty
CTX	5	≥ 20	17 – 19	< 17	
ETP	10	≥ 25	-	< 25	
GEN	10	≥ 17	-	< 17	
IPM	10	≥ 22	19 – 21	< 19	
MEM	10	≥ 22	16 – 21	< 16	
SXT	1.25 -23.75	≥ 14	11 – 13	< 11	
TZP	30 – 6	≥ 20	19**	< 20	** ATU – area of technical uncertainty

KLEBSIELLA

Additional Table 13. Antimicrobial resistance (R) in *Klebsiella* species from 2017-2020

Antimicrobial Agent	Antimicrobial Resistance % (R)				Overall Change (2017 vs 2020)
	2017	2018	2019	2020	
Amoxicillin-clavulanic acid (AMC)	45.11 %	43.43 %	35.91 %	47.20 %	↑
AMC_UTI	33.58 %	35.36 %	40.45 %	44.00 %	↑
Amikacin (AMK)	03.99 %	01.14 %	00.58 %	01.42 %	↓
Ampicillin (AMP)	100.00 %	99.80 %	100.00 %	99.67 %	↓
Ceftazidime (CAZ)	39.80 %	36.86 %	35.40 %	43.35 %	↑
Chloramphenicol (CHL)	20.52 %	16.28 %	12.36 %	05.88 %	↓
Ciprofloxacin (CIP)	43.07 %	36.19 %	35.71 %	40.98 %	↓
Cefotaxime (CTX)	39.65 %	37.04 %	35.05 %	44.92 %	↑
Ertapenem (ETP)	07.50 %	10.91 %	12.12 %	07.73 %	↑
Gentamicin (GEN)	34.94 %	33.14 %	33.15 %	24.53 %	↓
Imipenem (IPM)	01.26 %	00.56 %	00.56 %	01.61 %	↑
Meropenem (MEM)	01.26 %	00.37 %	00.56 %	01.59 %	↑
Trimethoprim-sulfamethoxazole (SXT)	30.56 %	31.98 %	32.63 %	42.93 %	↑
Piperacillin-tazobactam (TZP)	26.63 %	18.48 %	17.45 %	13.61 %	↓

* NT – not tested; UTI – uncomplicated urinary tract infection; ↑ indicates increase in resistance; ↓ indicates decrease in resistance; = indicates no change in resistance

Additional Table 14. Breakpoints for antibiotics against *Klebsiella* species in disk diffusion method (EUCAST guidelines v11.0; applicable Jan 2021)

Antibiotic	Disk Content (µg)	Susceptible (S) [in mm]		Resistant (R) [in mm]	Notes
		Susceptible, standard dosing (S)	Susceptible, increased exposure (I)		
AMC	10 – 20	≥ 19	-	< 19	
AMC_UTI	10 – 20	≥ 16	-	< 16	
AMK	30	≥ 18	-	< 18	
AMP	10	≥ 14		< 14	
CAZ	10	≥ 22	19 – 21	< 19	
CHL	30	≥ 17	-	< 17	
CIP	5	≥ 25	22 – 24**	< 22	** ATU – area of technical uncertainty
CTX	5	≥ 20	17 – 19	< 17	
ETP	10	≥ 25	-	< 25	
GEN	10	≥ 17	-	< 17	
IPM	10	≥ 22	19 – 21	< 19	
MEM	10	≥ 22	16 – 21	< 16	
SXT	1.25 -23.75	≥ 14	11 – 13	< 11	
TZP	30 – 6	≥ 20	19**	< 20	** ATU – area of technical uncertainty

MORAXELLA

Additional Table 15. Antimicrobial resistance (R) in *Moraxella* species from 2017-2020

Antimicrobial Agent	Antimicrobial Resistance % (R)				Overall Change (2018 vs 2020)
	2017	2018	2019	2020	
Amoxicillin-clavulanic acid (AMC)	NT	00.00 %	06.25 %	00.00 %	=
Chloramphenicol (CHL)	NT	00.00 %	00.00 %	00.00 %	=
Ciprofloxacin (CIP)	NT	00.00 %	00.00 %	00.00 %	=
Cefotaxime (CTX)	NT	00.00 %	00.00 %	00.00 %	=
Erythromycin (ERY)	NT	00.00 %	00.00 %	00.00 %	=
Tetracycline (TCY)	NT	00.00 %	00.00 %	00.00 %	=

* NT – not tested; UTI – uncomplicated urinary tract infection; ↑ indicates increase in resistance; ↓ indicates decrease in resistance; = indicates no change in resistance

Additional Table 16. Breakpoints for antibiotics against *Moraxella* species in disk diffusion method (EUCAST guidelines v11.0; applicable Jan 2021)

Antibiotic	Disk Content (μg)	Susceptible (S) [in mm]		Resistant (R) [in mm]	Notes
		Susceptible, standard dosing (S)	Susceptible, increased exposure (I)		
AMC	2 – 1	≥ 19	-	< 19	
CHL	30	≥ 31	-	< 31	For topical agent use only
CIP	5	≥ 31	-	< 31	
CTX	5	≥ 20	17 – 19	< 17	
ERY	15	≥ 23	20 – 22	< 20	
TCY	30	≥ 28	25 – 27	< 25	

MORGANELLA

Additional Table 17. Antimicrobial resistance (R) in *Morganella* species from 2017-2020

Antimicrobial Agent	Antimicrobial Resistance % (R)				Overall Change (2017 vs 2020)
	2017	2018	2019	2020	
Amoxicillin-clavulanic acid (AMC)	100.00 %	100.00 %	100.00 %	100.00 %	=
AMC_UTI	100.00 %	100.00 %	100.00 %	100.00 %	=
Amikacin (AMK)	00.00 %	00.00 %	00.00 %	00.00 %	=
Ampicillin (AMP)	100.00 %	100.00 %	100.00 %	100.00 %	=
Ceftazidime (CAZ)	03.13 %	12.24 %	16.28 %	15.56 %	↑
Chloramphenicol (CHL)	33.33 %	28.00 %	19.05 %	15.15 %	↓
Ciprofloxacin (CIP)	12.50 %	12.24 %	09.30 %	04.44 %	↓
Cefotaxime (CTX)	03.13 %	14.29 %	13.95 %	15.56 %	↑
Ertapenem (ETP)	00.00 %	02.04 %	00.00 %	00.00 %	=
Gentamicin (GEN)	06.25 %	08.16 %	04.65 %	00.00 %	↓
Imipenem (IPM)	00.00 %	00.00 %	00.00 %	00.00 %	=
Meropenem (MEM)	00.00 %	00.00 %	00.00 %	00.00 %	=
Trimethoprim-sulfamethoxazole (SXT)	21.88 %	27.08 %	18.60 %	15.56 %	↓
Piperacillin-tazobactam (TZP)	00.00 %	02.04 %	02.32 %	02.27 %	↑

* NT – not tested; UTI – uncomplicated urinary tract infection; ↑ indicates increase in resistance; ↓ indicates decrease in resistance; = indicates no change in resistance

Additional Table 18. Breakpoints for antibiotics against *Morganella* species in disk diffusion method (EUCAST guidelines v11.0; applicable Jan 2021)

Antibiotic	Disk Content (μg)	Susceptible (S) [in mm]		Resistant (R) [in mm]	Notes
		Susceptible, standard dosing (S)	Susceptible, increased exposure (I)		
AMC	10 – 20	≥ 19	19 – 20**	< 19	** ATU – area of technical uncertainty
AMC_UTI	10 – 20	≥ 16	-	< 16	
AMK	30	≥ 18	-	< 18	
AMP	10	≥ 14		< 14	
CAZ	10	≥ 22	19 – 21	< 19	
CHL	30	≥ 17	-	< 17	
CIP	5	≥ 25	22 – 24**	< 22	** ATU – area of technical uncertainty
CTX	5	≥ 20	17 – 19	< 17	
ETP	10	≥ 25	-	< 25	
GEN	10	≥ 17	-	< 17	
IPM	10	≥ 50	19 – 49	< 19	
MEM	10	≥ 22	16 – 21	< 16	
SXT	1.25 -23.75	≥ 14	11 – 13	< 11	
TZP	30-6	≥ 20	19**	< 20	** ATU – area of technical uncertainty

PROTEUS

Additional Table 19. Antimicrobial resistance (R) in *Proteus* species from 2017-2020

Antimicrobial Agent	Antimicrobial Resistance % (R)				Overall Change (2017 vs 2020)
	2017	2018	2019	2020	
Amoxicillin-clavulanic acid (AMC)	25.00 %	22.95 %	32.80 %	25.00 %	=
AMC_UTI	27.63 %	18.56 %	36.92 %	23.88 %	↓
Amikacin (AMK)	01.60 %	01.38 %	01.11 %	00.58 %	↓
Ampicillin (AMP)	65.93 %	55.07 %	64.94 %	59.52 %	↓
Ceftazidime (CAZ)	08.51 %	04.11 %	06.49 %	02.89 %	↓
Chloramphenicol (CHL)	34.23 %	44.07 %	45.00 %	46.73 %	↑
Ciprofloxacin (CIP)	37.57 %	31.31 %	48.91 %	38.01 %	↑
Cefotaxime (CTX)	26.74 %	18.81 %	40.54 %	23.84 %	↓
Ertapenem (ETP)	00.53 %	00.00 %	00.00 %	00.00 %	↓
Gentamicin (GEN)	31.18 %	28.57 %	47.51 %	30.64 %	↓
Imipenem (IPM)	00.00 %	00.00 %	00.00 %	00.00 %	=
Meropenem (MEM)	00.00 %	00.00 %	00.00 %	00.00 %	=
Trimethoprim-sulfamethoxazole (SXT)	49.46 %	41.59 %	58.99 %	45.03 %	↓
Piperacillin-tazobactam (TZP)	01.06 %	01.84 %	01.06 %	00.58 %	↓

* NT – not tested; UTI – uncomplicated urinary tract infection; ↑ indicates increase in resistance; ↓ indicates decrease in resistance; = indicates no change in resistance

Additional Table 20. Breakpoints for antibiotics against *Proteus* species in disk diffusion method (EUCAST guidelines v11.0; applicable Jan 2021)

Antibiotic	Disk Content (µg)	Susceptible (S) [in mm]		Resistant (R) [in mm]	Notes
		Susceptible, standard dosing (S)	Susceptible, increased exposure (I)		
AMC	10 – 20	≥ 19	19 – 20**	< 19	** ATU – area of technical uncertainty
AMC_UTI	10 – 20	≥ 16	-	< 16	
AMK	30	≥ 18	-	< 18	
AMP	10	≥ 14		< 14	
CAZ	10	≥ 22	19 – 21	< 19	
CHL	30	≥ 17	-	< 17	
CIP	5	≥ 25	22 – 24**	< 22	** ATU – area of technical uncertainty
CTX	5	≥ 20	17 – 19	< 17	
ETP	10	≥ 25	-	< 25	
GEN	10	≥ 17	-	< 17	
IPM	10	≥ 50	19 – 49	< 19	
MEM	10	≥ 22	16 – 21	< 16	
SXT	1.25 -23.75	≥ 14	11 – 13	< 11	
TZP	30-6	≥ 20	19**	< 20	** ATU – area of technical uncertainty

PSEUDOMONAS

Additional Table 21. Antimicrobial resistance (R) in *Pseudomonas* species from 2017-2020

Antimicrobial Agent	Antimicrobial Resistance % (R)				Overall Change (2017 vs 2020)
	2017	2018	2019	2020	
Amikacin (AMK)	05.42 %	01.62 %	02.03 %	02.19 %	↓
Ceftazidime (CAZ)	35.54 %	16.94 %	18.59 %	14.29 %	↓
Ciprofloxacin (CIP)	47.59 %	34.05 %	31.00 %	17.58 %	↓
Imipenem (IPM)	34.55 %	21.20 %	21.18 %	15.30 %	↓
Meropenem (MEM)	39.16 %	23.24 %	31.84 %	24.86 %	↓
Piperacillin-tazobactam (TZP)	43.03 %	27.81 %	25.37 %	21.31 %	↓

* NT – not tested; UTI – uncomplicated urinary tract infection; ↑ indicates increase in resistance; ↓ indicates decrease in resistance; = indicates no change in resistance

Additional Table 22. Breakpoints for antibiotics against *Pseudomonas* species in disk diffusion method (EUCAST guidelines v11.0; applicable Jan 2021)

Antibiotic	Disk Content (µg)	Susceptible (S) [in mm]		Resistant (R) [in mm]	Notes
		Susceptible, standard dosing (S)	Susceptible, increased exposure (I)		
AMK	30	≥ 15	-	< 15	
CAZ	10	≥ 50	17 – 49	< 17	
CIP	5	≥ 50	26 – 49	< 26	
IPM	10	≥ 50	20 – 49	< 20	
MEM	10	≥ 24	18 – 23	< 18	
TZP	30 – 6	≥ 50	19 – 49	< 18	**18 – 19 is ATU – area of technical uncertainty

SERRATIA

Additional Table 23. Antimicrobial resistance (R) in *Serratia* species from 2017-2020

Antimicrobial Agent	Antimicrobial Resistance % (R)				Overall Change (2017 vs 2020)
	2017	2018	2019	2020	
Amoxicillin-clavulanic acid (AMC)	100.00 %	100.00 %	100.00 %	100.00 %	=
AMC_UTI	100.00 %	100.00 %	100.00 %	100.00 %	=
Amikacin (AMK)	00.00 %	00.00 %	00.00 %	00.00 %	=
Ampicillin (AMP)	100.00 %	100.00 %	100.00 %	100.00 %	=
Ceftazidime (CAZ)	06.45 %	00.00 %	00.00 %	00.00 %	↓
Chloramphenicol (CHL)	07.69 %	02.22 %	05.26 %	00.00 %	↓
Ciprofloxacin (CIP)	03.23 %	07.69 %	04.54 %	02.33 %	↓
Cefotaxime (CTX)	06.45 %	09.43 %	04.65 %	02.33 %	↓
Ertapenem (ETP)	03.23 %	01.89 %	00.00 %	00.00 %	↓
Gentamicin (GEN)	06.45 %	05.88 %	02.33 %	00.00 %	↓
Imipenem (IPM)	00.00 %	00.00 %	00.00 %	00.00 %	=
Meropenem (MEM)	03.23 %	00.00 %	00.00 %	00.00 %	↓
Trimethoprim-sulfamethoxazole (SXT)	10.00 %	01.92 %	04.76 %	04.65 %	↓
Piperacillin-tazobactam (TZP)	06.45 %	03.77 %	02.33 %	00.00 %	↓

* NT – not tested; UTI – uncomplicated urinary tract infection; ↑ indicates increase in resistance; ↓ indicates decrease in resistance; = indicates no change in resistance

Additional Table 24. Breakpoints for antibiotics against *Serratia* species in disk diffusion method (EUCAST guidelines v11.0; applicable Jan 2021)

Antibiotic	Disk Content (µg)	Susceptible (S) [in mm]		Resistant (R) [in mm]	Notes
		Susceptible, standard dosing (S)	Susceptible, increased exposure (I)		
AMC	10 – 20	≥ 19	-	< 19	
AMC_UTI	10 – 20	≥ 16	-	< 16	
AMK	30	≥ 18	-	< 18	
AMP	10	≥ 14	-	< 14	
CAZ	10	≥ 22	19 – 21	< 19	
CHL	30	≥ 17	-	< 17	
CIP	5	≥ 25	22 – 24**	< 22	** ATU – area of technical uncertainty
CTX	5	≥ 20	17 – 19	< 17	
ETP	10	≥ 25	-	< 25	
GEN	10	≥ 17	-	< 17	
IPM	10	≥ 22	19 – 21	< 19	
MEM	10	≥ 22	16 – 21	< 16	
SXT	1.25 -23.75	≥ 14	11 – 13	< 11	
TZP	30 – 6	≥ 20	19**	< 20	** ATU – area of technical uncertainty

STENOTROPHOMONAS

Additional Table 25. Antimicrobial resistance (R) in *Stenotrophomonas* species from 2017-2020

Antimicrobial Agent	Antimicrobial Resistance % (R)				Overall Change (2017 vs 2020)
	2017	2018	2019	2020	
Trimethoprim-sulfamethoxazole (SXT)	08.69 %	03.64 %	02.35 %	00.00 %	↓

* NT – not tested; UTI – uncomplicated urinary tract infection; ↑ indicates increase in resistance; ↓ indicates decrease in resistance; = indicates no change in resistance

Additional Table 26. Breakpoints for antibiotics against *Stenotrophomonas* species in disk diffusion method (EUCAST guidelines v11.0; applicable Jan 2021)

Antibiotic	Disk Content (µg)	Susceptible (S) [in mm]		Resistant (R) [in mm]	Notes
		Susceptible, standard dosing (S)	Susceptible, increased exposure (I)		
SXT	1.25 -23.75	≥ 50	16 – 49	< 16	
