# Supplementary material for: Antimicrobial Resistance in Nosocomial Isolates of Gram-Negative Bacteria: Public Health Implications in the Latvian Context
Source: Antibiotics (Basel). 2021 Jun 29;10(7):791. doi: 10.3390/antibiotics10070791 (PMC8300747; doi:10.3390/antibiotics10070791)
Supplement: Supplementary file 1 [file antibiotics-10-00791-s001.zip › Supplementary File S3.pdf]

ADDITIONAL FILE 3
MULTIPLE ANTIBIOTIC RESISTANCE (MAR) INDEX RESULTS

Antimicrobial Resistance in Nosocomial Isolates of Gram-Negative Bacteria: Public Health Implications in Latvian Context

Authors: Nityanand Jain*, Inese Jansone, Tatjana Obidenova, Raimonds Sīmanis, Jānis Meisters, Dagnija Straupmane and Aigars Reinis

*** For Correspondence:**

Department of Biology and Microbiology
Riga Stradins University
16 Dzirciema street
Riga
Latvia (LV-1007)

Email: nityapkl@gmail.com

INDEX

1) Additional Table 1 – MAR Index in *Acinetobacter* species
2) Additional Table 2 – MAR Index in *Aeromonas* species
3) Additional Table 3 – MAR Index in *Burkholderia* species
4) Additional Table 4 – MAR Index in *Citrobacter* species
5) Additional Table 5 – MAR Index in *Enterobacter* species
6) Additional Table 6 – MAR Index in *Escherichia* species
7) Additional Table 7 – MAR Index in *Klebsiella* species
8) Additional Table 8 – MAR Index in *Moraxella* species
9) Additional Table 9 – MAR Index in *Morganella* species
10) Additional Table 10 – MAR Index in *Proteus* species
11) Additional Tables 11 – MAR Index in *Pseudomonas* species
12) Additional Tables 12 – MAR Index in *Serratia* species

ACINETOBACTER

Additional Table 1. Multiple antibiotic resistance (MAR) Index in *Acinetobacter* species from 2017-2020

No. of Antimicrobial agents	2017		2020		Overall Change in % of Isolates
	% of Isolates	MAR Index	% of Isolates	MAR Index	
5	-	-	-	-	-
4	79.00 %	0.8	30.53 %	0.8	↓
3	01.33 %	0.6	27.48 %	0.6	↑
2	02.00 %	0.4	11.07 %	0.4	↑
1	07.00 %	0.2	04.58 %	0.2	↓
No resistance against any tested agent (0)	10.67 %	0.0	26.37 %	0.0	↑

* ↑ indicates increase in % of MAR isolates; ↓ indicates decrease in % of MAR isolates; = indicates no change in resistance

Interpretation – The overall MDR rates (resistance to ≥ 2 antimicrobial agents) in 2017 amongst the *Acinetobacter* isolates were 82.33 % which dropped to 69.08 % in 2020.

AEROMONAS

Additional Table 2. Multiple antibiotic resistance (MAR) Index in *Aeromonas* species from 2018-2020

No. of Antimicrobial agents	2018		2020		Overall Change in % of Isolates
	% of Isolates	MAR Index	% of Isolates	MAR Index	
3	-	-	-	-	-
2	-	-	-	-	-
1	33.33 %	0.3	25.00 %	0.3	↓
No resistance against any tested agent (0)	66.66 %	0.0	75.00 %	0.0	↑

* ↑ indicates increase in % of MAR isolates; ↓ indicates decrease in % of MAR isolates; = indicates no change in resistance

Interpretation – The overall MDR rates (resistance to ≥ 2 antimicrobial agents) in 2017 amongst the *Aeromonas* isolates were 00.00 % which remained the same in 2020.

BURKHOLDERIA

Additional Table 3. Multiple antibiotic resistance (MAR) Index in *Burkholderia* species from 2017-2020

**No. of Antimicrobial agents**	**2017**		**2020**		**Overall Change in % of Isolates**
	**% of Isolates**	**MAR Index**	**% of Isolates**	**MAR Index**	
4	-	-	-	-	-
3	-	-	-	-	-
2	-	-	-	-	-
1	-	-	-	-	-
No resistance against any tested agent (0)	100.00 %	0.0	100.00 %	0.0	=

* ↑ indicates increase in % of MAR isolates; ↓ indicates decrease in % of MAR isolates; = indicates no change in resistance

Interpretation – The overall MDR rates (resistance to ≥ 2 antimicrobial agents) in 2017 amongst the *Burkholderia* isolates were 00.00 % which remained the same in 2020.

CITROBACTER

Additional Table 4. Multiple antibiotic resistance (MAR) Index in *Citrobacter* species from 2017-2020

No. of Antimicrobial agents	2017		2020		Overall Change in % of Isolates
	% of Isolates	MAR Index	% of Isolates	MAR Index	
13	-	-	-	-	-
12	-	-	-	-	-
11	-	-	-	-	-
10	01.61 %	0.8	00.00 %	0.8	↓
9	00.00 %	0.7	00.00 %	0.7	↓
8	01.61 %	0.6	04.35 %	0.6	↑
7	03.23 %	0.5	02.90 %	0.5	↓
6	11.29 %	0.5	01.45 %	0.5	↓
5	04.84 %	0.4	07.25 %	0.4	↑
4	08.06 %	0.3	13.04 %	0.3	↑
3	03.23 %	0.2	07.25 %	0.2	↑
2	32.26 %	0.2	37.68 %	0.2	↑
1	33.87 %	0.1	24.64 %	0.1	↓
No resistance against any tested agent (0)	00.00 %	0.0	01.45 %	0.0	↑

* ↑ indicates increase in % of MAR isolates; ↓ indicates decrease in % of MAR isolates; = indicates no change in resistance

Interpretation – The overall MDR rates (resistance to ≥ 2 antimicrobial agents) in 2017 amongst the *Citrobacter* isolates were 66.13 % which increased to 73.91 % in 2020.

ENTEROBACTER

Additional Table 5. Multiple antibiotic resistance (MAR) Index in *Enterobacter* species from 2017-2020

No. of Antimicrobial agents	2017		2020		Overall Change in % of Isolates
	% of Isolates	MAR Index	% of Isolates	MAR Index	
13	-	-	-	-	-
12	-	-	-	-	-
11	-	-	-	-	-
10	-	-	-	-	-
9	01.17 %	0.7	00.00 %	0.7	↓
8	11.70 %	0.6	04.33 %	0.6	↓
7	07.02 %	0.5	05.63 %	0.5	↓
6	12.28 %	0.5	09.96 %	0.5	↓
5	07.02 %	0.4	14.72 %	0.4	↑
4	02.34 %	0.3	06.49 %	0.3	↑
3	05.85 %	0.2	04.76 %	0.2	↓
2	44.44 %	0.2	43.29 %	0.2	↓
1	08.19 %	0.1	10.82 %	0.1	↑
No resistance against any tested agent (0)	-	-	-	-	-

* ↑ indicates increase in % of MAR isolates; ↓ indicates decrease in % of MAR isolates; = indicates no change in resistance

Interpretation – The overall MDR rates (resistance to ≥ 2 antimicrobial agents) in 2017 amongst the *Enterobacter* isolates were 91.81 % which decreased to 89.18 % in 2020.

ESCHERICHIA

Additional Table 6. Multiple antibiotic resistance (MAR) Index in *Escherichia* species from 2017-2020

No. of Antimicrobial agents	2017		2020		Overall Change in % of Isolates
	% of Isolates	MAR Index	% of Isolates	MAR Index	
13	-	-	-	-	-
12	-	-	-	-	-
11	-	-	-	-	-
10	-	-	-	-	-
9	00.21 %	0.7	00.27 %	0.7	↑
8	00.53 %	0.6	01.07 %	0.6	↑
7	01.71 %	0.5	02.28 %	0.5	↑
6	03.84 %	0.5	04.02 %	0.5	↑
5	04.06 %	0.4	05.23 %	0.4	↑
4	03.74 %	0.3	06.17 %	0.3	↑
3	05.87 %	0.2	08.85 %	0.2	↑
2	10.57 %	0.2	14.21 %	0.2	↑
1	17.08 %	0.1	17.02 %	0.1	↓
No resistance against any tested agent (0)	52.40 %	0.0	40.88 %	0.0	↓

* ↑ indicates increase in % of MAR isolates; ↓ indicates decrease in % of MAR isolates; = indicates no change in resistance

Interpretation – The overall MDR rates (resistance to ≥ 2 antimicrobial agents) in 2017 amongst the *Escherichia* isolates were 30.53 % which increased to 42.09 % in 2020.

KLEBSIELLA

Additional Table 7. Multiple antibiotic resistance (MAR) Index in *Klebsiella* species from 2017-2020

No. of Antimicrobial agents	2017		2020		Overall Change in % of Isolates
	% of Isolates	MAR Index	% of Isolates	MAR Index	
13	-	-	-	-	-
12	-	-	-	-	-
11	00.52 %	0.8	01.70 %	0.8	↑
10	01.04 %	0.8	00.85 %	0.8	↓
9	03.91 %	0.7	01.36 %	0.7	↓
8	07.03 %	0.6	05.11 %	0.6	↓
7	11.72 %	0.5	10.56 %	0.5	↓
6	11.46 %	0.5	16.18 %	0.5	↑
5	04.17 %	0.4	08.52 %	0.4	↑
4	02.60 %	0.3	02.56 %	0.3	↓
3	02.60 %	0.2	03.07 %	0.2	↑
2	06.25 %	0.2	06.13 %	0.2	↓
1	48.70 %	0.1	43.61 %	0.1	↓
No resistance against any tested agent (0)	00.00 %	0.0	00.34 %	0.0	↑

* ↑ indicates increase in % of MAR isolates; ↓ indicates decrease in % of MAR isolates; = indicates no change in resistance

Interpretation – The overall MDR rates (resistance to ≥ 2 antimicrobial agents) in 2017 amongst the *Klebsiella* isolates were 51.30 % which increased to 56.05 % in 2020.

MORAXELLA

Additional Table 8. Multiple antibiotic resistance (MAR) Index in *Moraxella* species from 2018-2020

No. of Antimicrobial agents	2018		2020		Overall Change in % of Isolates
	% of Isolates	MAR Index	% of Isolates	MAR Index	
6	-	-	-	-	-
5	-	-	-	-	-
4	-	-	-	-	-
3	-	-	-	-	-
2	-	-	-	-	-
1	-	-	-	-	-
No resistance against any tested agent (0)	100.00 %	0.0	100.00 %	0.0	=

* ↑ indicates increase in % of MAR isolates; ↓ indicates decrease in % of MAR isolates; = indicates no change in resistance

Interpretation – The overall MDR rates (resistance to ≥ 2 antimicrobial agents) in 2017 amongst the *Moraxella* isolates were 0.00 % which remained the same in 2020.

MORGANELLA

Additional Table 9. Multiple antibiotic resistance (MAR) Index in *Morganella* species from 2017-2020

No. of Antimicrobial agents	2017		2020		Overall Change in % of Isolates
	% of Isolates	MAR Index	% of Isolates	MAR Index	
13	-	-	-	-	-
12	-	-	-	-	-
11	-	-	-	-	-
10	-	-	-	-	-
9	-	-	-	-	-
8	-	-	-	-	-
7	-	-	-	-	-
6	03.13 %	0.5	00.00 %	0.5	↓
5	03.13 %	0.4	06.82 %	0.4	↑
4	15.63 %	0.3	11.36 %	0.3	↓
3	18.75 %	0.2	15.91 %	0.2	↓
2	37.50 %	0.2	50.00 %	0.2	↑
1	21.88 %	0.1	15.91 %	0.1	↓
No resistance against any tested agent (0)	-	-	-	-	-

* ↑ indicates increase in % of MAR isolates; ↓ indicates decrease in % of MAR isolates; = indicates no change in resistance

Interpretation – The overall MDR rate (resistance to ≥ 2 antimicrobial agents) in 2017 amongst the *Morganella* isolates were 78.12 % which increased to 84.09 % in 2020.

PROTEUS

Additional Table 10. Multiple antibiotic resistance (MAR) Index in *Proteus* species from 2017-2020

No. of Antimicrobial agents	2017		2020		Overall Change in % of Isolates
	% of Isolates	MAR Index	% of Isolates	MAR Index	
13	-	-	-	-	-
12	-	-	-	-	-
11	-	-	-	-	-
10	-	-	-	-	-
9	-	-	-	-	-
8	02.22 %	0.6	00.60 %	0.6	↓
7	03.33 %	0.5	09.58 %	0.5	↑
6	05.56 %	0.5	00.60 %	0.5	↓
5	13.33 %	0.4	07.19 %	0.4	↓
4	04.44 %	0.3	08.38 %	0.3	↑
3	05.56 %	0.2	08.38 %	0.2	↑
2	14.44 %	0.2	10.18 %	0.2	↓
1	16.11 %	0.1	14.97 %	0.1	↓
No resistance against any tested agent (0)	35.00 %	0.0	40.12 %	0.0	↑

* ↑ indicates increase in % of MAR isolates; ↓ indicates decrease in % of MAR isolates; = indicates no change in resistance

Interpretation – The MDR rate (resistance to ≥ 2 antimicrobial agents) in 2017 amongst the *Proteus* isolates were 48.89 % which decreased to 44.91 % in 2020.

PSEUDOMONAS

Additional Table 11. Multiple antibiotic resistance (MAR) Index in *Pseudomonas* species from 2017-2020

No. of Antimicrobial agents	2017		2020		Overall Change in % of Isolates
	% of Isolates	MAR Index	% of Isolates	MAR Index	
6	03.09 %	1.0	01.46 %	1.0	↓
5	17.90 %	0.8	01.46 %	0.8	↓
4	04.32 %	0.7	02.43 %	0.7	↓
3	04.94 %	0.5	02.43 %	0.5	↓
2	07.41 %	0.3	09.71 %	0.3	↑
1	09.88 %	0.2	07.28 %	0.2	↓
No resistance against any tested agent (0)	52.47 %	0.0	75.24 %	0.0	↑

* ↑ indicates increase in % of MAR isolates; ↓ indicates decrease in % of MAR isolates; = indicates no change in resistance

Interpretation – The overall MDR rate (resistance to ≥ 2 antimicrobial agents) in 2017 amongst the *Pseudomonas* isolates were 37.65 % which decreased to 17.48 % in 2020.

SERRATIA

Additional Table 12. Multiple antibiotic resistance (MAR) Index in *Serratia* species from 2017-2020

No. of Antimicrobial agents	2017		2020		Overall Change in % of Isolates
	% of Isolates	MAR Index	% of Isolates	MAR Index	
13	-	-	-	-	-
12	-	-	-	-	-
11	-	-	-	-	-
10	-	-	-	-	-
9	03.23 %	0.7	00.00 %	0.7	↓
8	03.23 %	0.6	00.00 %	0.6	↓
7	00.00 %	0.5	00.00 %	0.5	↓
6	00.00 %	0.5	00.00 %	0.5	↓
5	00.00 %	0.4	00.00 %	0.4	↓
4	03.23 %	0.3	00.00 %	0.3	↓
3	03.23 %	0.2	04.76 %	0.2	↑
2	74.19 %	0.2	80.95 %	0.2	↑
1	12.90 %	0.1	14.29 %	0.1	↑
No resistance against any tested agent (0)	-	-	-	-	-

* ↑ indicates increase in % of MAR isolates; ↓ indicates decrease in % of MAR isolates; = indicates no change in resistance

Interpretation – The overall MDR rate (resistance to ≥ 2 antimicrobial agents) in 2017 amongst the *Serratia* isolates were 87.10 % which decreased marginally to 85.71 % in 2020.
